# Determining and controlling conformational information from orientationally selective light-induced triplet–triplet electron resonance spectroscopy for a set of bis-porphyrin rulers†

**DOI:** 10.1039/d3cp03454b

**Published:** 2024-01-03

**Authors:** Arnau Bertran, Marta De Zotti, Christiane R. Timmel, Marilena Di Valentin, Alice M. Bowen

**Affiliations:** a Centre for Advanced Electron Spin Resonance and Inorganic Chemistry Laboratory, Department of Chemistry, University of Oxford South Parks Road Oxford OX1 3QR UK arnau.bertran@chem.ox.ac.uk; b Department of Chemical Sciences, University of Padova Via Marzolo 1 35131 Padova Italy; c Centro Interdipartimentale di Ricerca “Centro Studi di Economia e Tecnica dell’energia Giorgio Levi Cases” 35131 Padova Italy marilena.divalentin@unipd.it; d The National Research Facility for Electron Paramagnetic Resonance, Department of Chemistry and Photon Science Institute, The University of Manchester Oxford Road Manchester M13 9PL UK alice.bowen@manchester.ac.uk

## Abstract

We recently reported a new technique, light-induced triplet–triplet electron resonance (LITTER) spectroscopy, which allows quantification of the dipolar interaction between the photogenerated triplet states of two chromophores. Here we carry out a systematic LITTER study, considering orientation selection by the detection pulses, of a series of bis-porphyrin model peptides with different porphyrin–porphyrin distances and relative orientations. Orientation-dependent analysis of the dipolar datasets yields conformational information of the molecules in frozen solution which is in good agreement with density functional theory predictions. Additionally, a fast partial orientational-averaging treatment produces distance distributions with minimized orientational artefacts. Finally, by direct comparison of LITTER data to double electron–electron resonance (DEER) measured on a system with Cu(ii) coordinated into the porphyrins, we demonstrate the advantages of the LITTER technique over the standard DEER methodology. This is due to the remarkable spectroscopic properties of the photogenerated porphyrin triplet state. This work sets the basis for the use of LITTER in structural investigations of unmodified complex biological macromolecules, which could be combined with Förster resonance energy transfer and microscopy inside cells.

## Abstract 2

We demonstrate light-induced triplet–triplet electron resonance spectroscopy applied to bis-porphyrin model peptides is a powerful technique to provide information on the relative chromophore positions.

## Graphical abstract

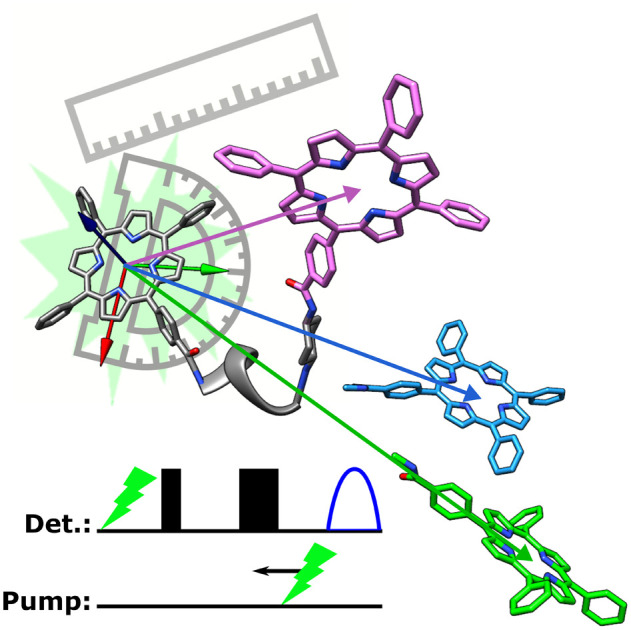

## Introduction

**Fig. 1:**
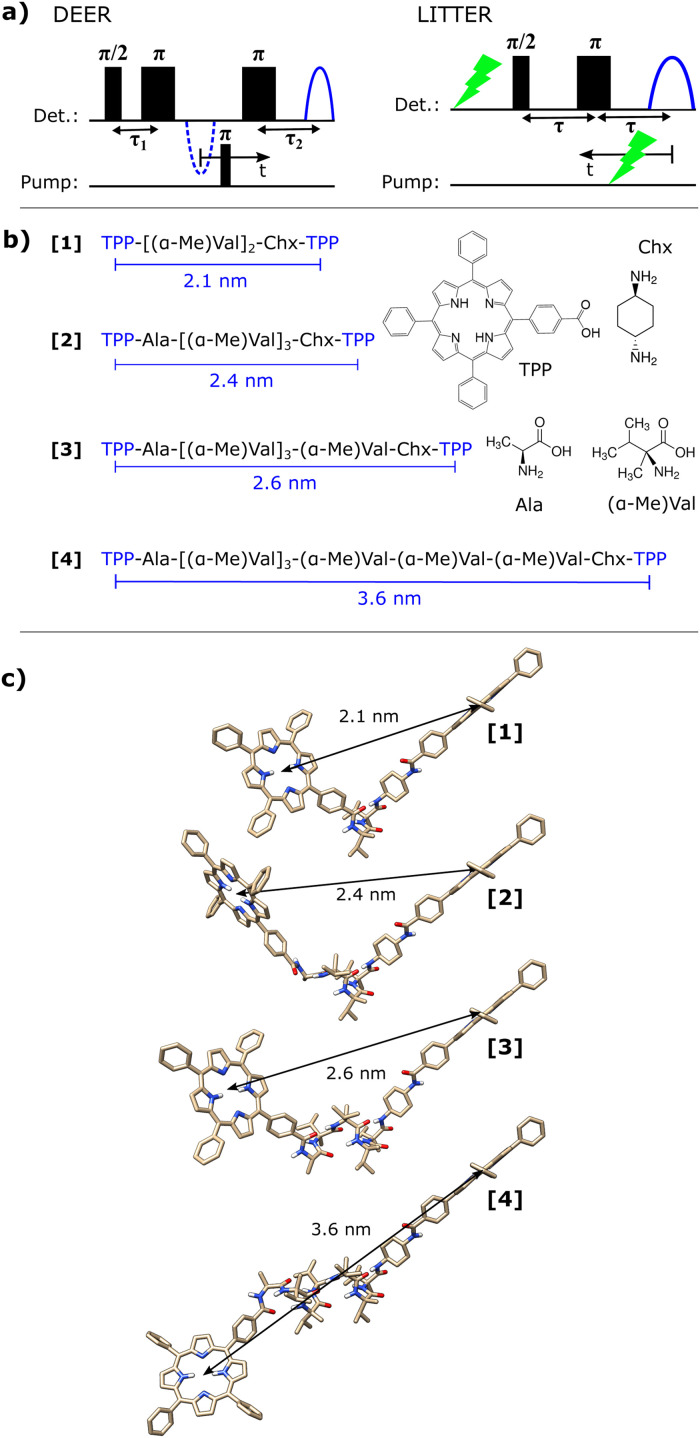
(a) Pulse sequences for DEER (left) and LITTER (right), with time axes indicated. (b) Amino acid sequences for the bis-porphyrin synthetic model peptides used in this study, with the porphyrin–porphyrin distances predicted by DFT. The chemical structures of the different building blocks are shown on the right. Amino acid key: Ala (l-alanine), (αMe)Val (l-α-methyl valine) and Chx (*trans*-1,4-diaminocyclohexane). (c) *In-vacuo* DFT-optimized geometries of the molecules.

The structural and dynamical study of complex biological macromolecular systems is of great interest in physical and life sciences.^1^ Electron spin resonance (ESR) pulsed dipolar spectroscopy (PDS) allows for the determination of the distribution of distances and, in some cases, relative orientations between two paramagnetic centers by measuring their electron–electron dipolar interaction, providing valuable conformational information on the system containing the paramagnetic species.^2^ The well-established PDS technique double electron–electron resonance,^3^ (DEER, Fig. 1(a), left) gives access to the distance range between 1.4 to more than 8 nm, very relevant for biological structural determination.^4^ The methodology for orientation selection in DEER of rigid and semi-rigid systems is well established and has included measurements on tyrosyl radicals,^5^ triarylmethyl radicals,^6^ rigid nucleotides in DNA and RNA,^7,8^ and metal centers and clusters in biological systems,^9,10^ amongst other examples.

Modified versions of the DEER technique have been developed using the photogenerated triplet state (*S* = 1) of a 5(4′-carboxyphenyl)-10,15,20-triphenylporphyrin moiety (TPP) as a photoswitchable spin label, formed by laser excitation during the PDS experiment.^11–16^ This photogenerated triplet state has the advantage, with respect to conventional spin labels, that it is formed in a spin polarized state, with a non-Boltzmann population of the triplet state sublevels. These populations are a result of the intersystem crossing (ISC) process and provide enhanced ESR sensitivity.^17^ In combination with a nitroxide radical, the photogenerated triplet state of a TPP moiety formed by a laser flash preceding the microwave (MW) pulse sequence was used as detection spin in light-induced DEER (LiDEER), allowing for the determination of TPP–nitroxide distances between 1.8 to 8.1 nm in a synthetic model peptide ruler, in agreement with conventional Cu(ii)–nitroxide DEER, measured on the same systems with Cu(ii) coordinated to the TPP moieties and corresponding density functional theory (DFT) calculations.^11,18^ Alternatively, the MW pump pulse of the DEER experiment was replaced by a variable-time laser flash to form the photogenerated TPP triplet state and switch on its dipolar interaction with a nitroxide radical in a synthetic model peptide, which was used as detection spin, in laser-induced magnetic dipole (LaserIMD) spectroscopy.^12^ Hahn-echo detection and refocused-echo detection versions of the LaserIMD technique were successfully used for distance measurements in porphyrin-containing proteins labelled with nitroxides,^14,19,20^ and were extended to photogenerated triplet states of other chromophores.^21–24^

Additional structural information, concerning the relative orientation between the porphyrin and the nitroxide moieties, has been obtained in rigid synthetic model peptides, where there was a strong correlation between the orientation of the most anisotropic magnetic tensor frame of the detection spin center and the angle of the spin–spin dipolar vector with the externally applied static magnetic field.^25^ Orientation selection by narrow bandwidth MW pulses with respect to the ESR spectrum of the detection spin center was exploited to obtain orientation-resolved LiDEER and LaserIMD datasets, yielding information on the conformational distribution of the molecule in a frozen solution. Orientationally selective LiDEER can be advantageous over orientation-resolved DEER using permanent spin centres,^26^ as the ZFS origin of the orientation selection removes the need for high fields.^27–29^ Photogenerated triplet states also have more favorable spectroscopic properties, as a detection species, compared to the *g*-anisotropic metal centers commonly used to achieve high orientational resolution,^30^ namely non-Boltzmann spin sublevel population and slower spin relaxation.^15,16^ Additionally, the single-frequency nature of LaserIMD, as also used in relaxation-induced dipolar modulation enhancement (RIDME)^31,32^ or the single-frequency technique for refocusing dipolar couplings (SIFTER),^33^ eliminates the limitation in pump-detection frequency offset due to limited resonator bandwidth.

We recently presented a new light-induced PDS technique, light-induced triplet–triplet electron resonance (LITTER) spectroscopy (Fig. 1(a), right),^34^ which enables the measurement of the dipolar interaction between two chromophores with photogenerated triplet states by combining the ideas of both LiDEER and LaserIMD: a photogenerated triplet state formed by a first laser flash preceding the MW Hahn echo pulse sequence is used as detection spin center, while a second variable-time laser flash is used to form the second triplet, switching on the dipolar interaction between the two spin centers. Starting with the second laser occurring after the spin echo and moving the position of the MW pulses in time relative to this laser (Fig. 1(a), right), the zero time of the dipolar trace occurs when the laser flash crosses the center of the echo. A dipolar trace of up to *τ* in length can be acquired by scanning the second laser between the echo and the MW π pulse. A symmetric image of this trace is obtained if the second laser continues to move until reaching the MW π/2 pulse. However, upon crossing of the first π/2 pulse in the MW pulse sequence and the second laser pulse, a large step in echo intensity occurs as the triplets formed by both laser flashes are now being refocused by the Hahn echo MW pulse sequence, and detected as part of the echo signal, severely distorting the start of the dipolar modulation. For this reason, when a symmetric trace is not required, the experiment is better carried out with the second laser moving between the echo and the π pulse. It has been shown for LaserIMD that the symmetry of the trace can be used to help define the zero time of the modulation, however this can also be more easily defined using a refocused Hahn echo sequence. A similar detection sequence could be applied in the LITTER experiment, but would result in a decrease in signal-to-noise ratio of the data collected due to a decrease in the measured echo intensity.^14^

LITTER benefits from being a single-frequency experiment with effectively an unlimited pump bandwidth afforded by using a laser flash to form the second triplet, during a simple Hahn-echo detection sequence. In addition, it removes the need of having at least one permanent spin center to perform PDS, allowing chromophore-containing macromolecules, which are ESR-silent in their ground state, to be studied. This could be advantageous for in-cell applications, where conventional nitroxide spin labels suffer from limited stability,^35,36^ and could facilitate the study of biological macromolecular systems, without the need for spin labelling, as triplet states of intrinsic chromophores or cofactors could be used as spin centers.

The first proof-of-concept LITTER work was carried out on a single bis-porphyrin synthetic model peptide, containing two identical TPP chromophores.^34^ Both laser pulses used the same wavelength, matching the absorption maximum of TPP in the visible. The dipolar modulation of the TPP triplet electron spin echo intensity relied on the statistical excitation of only one of the two porphyrins by the first laser followed by the excitation of the second porphyrin of the same molecule by the second laser. Consequently, the maximum modulation depth was limited for this system. Comparison to a control model peptide, containing a single TPP, for which no modulation of the echo intensity was observed, was used to prove that the modulation obtained with the bis-porphyrin peptide was the result of a true intramolecular triplet–triplet dipolar interaction.

Here we perform an extensive orientational LITTER study of a series of bis-porphyrin synthetic model peptide systems with different porphyrin–porphyrin relative orientations and distances ranging between 2.1 and 3.6 nm (Fig. 1(b) and (c)) to investigate the general performance and limitations of the LITTER technique. For each molecule, multiple orientationally selective traces are acquired at different parts of the porphyrin triplet ESR spectrum, capturing detailed information on the relative orientation of the ZFS tensor and the dipolar vector. The orientation-dependent analysis of these traces yields information on the conformation of the molecules in frozen solution which is in good agreement with the minimum energy geometries predicted by DFT. In addition, we perform a direct comparison between LITTER and DEER, by introducing Cu(ii) in the two porphyrins of one of our synthetic model peptides. Finally, we show an alternative treatment of the orientationally selective data, partially averaging the orientational effects in the dipolar traces to facilitate a computationally faster orientation-independent analysis. We compare these results to those obtained from the full orientation-dependent treatment.

## Experimental details

### Synthesis

Molecules [1], [2], [3] and [4] were synthesized as previously reported.^37,38^ Cu_2_-[3] was prepared by stirring a solution of [3] in d_6_-ethanol (99% atom, Aldrich) at room temperature overnight in an excess of Cu(ii) acetate (98%, Aldrich). The complete metalation of the sample was verified by UV-Vis (Fig. S4, ESI†).

### Sample preparation

Samples for light-induced ESR were prepared at a molecular concentration of ∼40 μM in d_6_-ethanol, and were loaded in 4 mm diameter quartz tubes to a sample height of ∼5 mm. The solvent was chosen to improve solubility and favour helicity of the peptide backbones. Samples were degassed by several freeze–pump–thaw cycles and frozen in liquid nitrogen prior to insertion into the spectrometer. In the case of molecule [4], some aggregation by porphyrin stacking, evidenced by the sample turning green during the freeze–pump–thaw process, was observed. For this reason, samples of [4] were prepared by dissolving the solid in previously degassed solvent, and were not degassed any further. The less effective O_2_ removal from this sample resulted in slightly shorter *T*_m_ values (Table S1, ESI†) but did not lead to any observable increase in photobleaching.

### ESR spectroscopy

Light-induced ESR experiments with molecules [1]–[4] were performed at X-band (microwave frequency = 9.7 GHz) and a temperature of 20 K in a pulsed spectrometer (ElexSys E680, Bruker) using a dielectric resonator (EN 4118XMD5, Bruker) and an Oxford Instruments cryostat. The two-laser setup was as previously reported.^34^ Laser 1 (OPOlette, Lambda Photometrics) was directed into the resonator through the optical window of the cryostat, while laser 2 (VersaScan OPO, GWU) was delivered *via* a 1 mm × 6 m optical fiber (FT1000-EMT, Thorlabs) directly inserted inside the sample tube. Both lasers were set at a wavelength of 512 nm, corresponding to the most intense maximum of the porphyrin Q-band region determined by UV-Vis (Fig. S1, ESI†), and 2–3 mJ per flash. For the pulse experiments, a delay pulse generator was used to externally trigger both lasers and the spectrometer at a repetition rate of 20 Hz, and the microwave pulses were moved forward in time with respect to the fixed laser flashes. All spectra were acquired with one shot per point.

Time-resolved ESR (trESR) was carried out in a critically coupled resonator using Laser 1 only, without field modulation or phase sensitive detection. The signal was averaged around the intensity maximum of the time trace. The spin Hamiltonian parameters for the photoexcited triplet state were extracted *via* simulation of the spectrum (Fig. S2, ESI†) using the Matlab® EasySpin routine (pepper function).^39^

Light-induced pulsed ESR characterization (*i.e.* field sweeps, phase-memory time and delay-after-flash experiments) were performed in an over-coupled resonator using a standard Hahn-echo sequence preceded by a laser flash (laser 1-DAF-π/2–*τ*–π–*τ*-echo), with 16–32 ns π/2–π rectangular pulses.

LITTER traces were acquired using the following pulse sequence: laser 1-DAF-π/2–*τ*–π–*τ*′-laser 2-*τ*′′-echo, *τ* = *τ*′ + *τ*′′, with 16–32 ns π/2–π rectangular pulses. The delay between flashes was *t*_pp_ = 7 μs. Measurements were carried out at different values of the external magnetic field, and the exact *τ* delays were adjusted to match electron spin envelope modulation (ESEEM) maxima observed in the phase-memory-time experiments at the corresponding field positions (Fig. S3(a), ESI†). Raw LITTER traces were phase- and background-corrected before analysis. Modulation depths were very sensitive to the power fluctuations of laser 2, solvent glass cracking and sample bleaching, leading to slightly different modulation depths for different traces. For this reason, modulation depths were normalized to 1 before analysis.

ESR experiments with Cu_2_-[3] were performed at Q-band (microwave frequency = 34 GHz) and a temperature of 15 K in a pulsed spectrometer (ElexSys E580, Bruker) using a dielectric resonator (EN 5107D2, Bruker) and an Oxford Instruments cryostat. Field sweeps and phase-memory time experiments were performed in an over-coupled resonator using a standard Hahn-echo sequence (π/2–*τ*–π–*τ*–echo), with 16–32 ns π/2–π rectangular pulses. For inversion-recovery experiments, this sequence was preceded by an inversion pulse (π–T–π/2–*τ*–π–*τ*–echo). The spin Hamiltonian parameters for the molecule were extracted *via* simulation of the field-swept spectrum (Fig. S2, ESI†) using the Matlab® EasySpin routine (pepper function).^39^

DEER traces were acquired at different external field values using the 4-pulse refocused-echo version of the experiment, shown in Fig. 1(a): π/2–*τ*_1_–π–*τ*′–πpump–*τ*′′–π–*τ*_2_– echo, *τ*_1_ = 200 ns, *τ*_2_ = 2000 ns, *τ*1 + *τ*2 = *τ*′ + *τ*′′, with 16 ns rectangular detection pulses and a 12 ns rectangular pump pulse. The pump frequency was set at −100 MHz from the detection frequency. The pump frequency was set to be at the centre of the resonator bandwidth. A 16-step phase cycle and 8-step *τ*_1_- and *τ*_2_- averaging cycles were used. The experiment was repeated at a rate of 500 Hz, with 20 shots per point. Raw DEER traces were phase- and background-corrected before analysis.

For the orientation-independent analysis of LITTER and DEER, the dipolar traces acquired at different field positions were averaged weighted by the corresponding spectral intensities to obtain an orientation-independent form factor, which was then analysed *via* Fourier Transform and Tikhonov regularization using the Matlab® DeerAnalysis routine to extract the corresponding distance distribution.^51^ A *g*-factor correction was applied to the distance distribution, as previously described,^41^ using the following expression
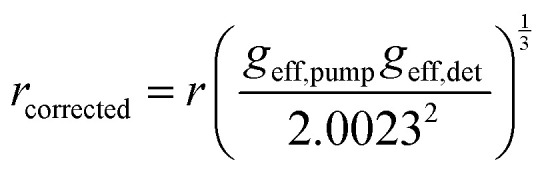
where *g*_eff_,_pump_ and *g*_eff_,_det_ are the effective *g*-values of the pump and detection spin centres, calculated as averages weighted by the corresponding spectral intensities.

The metadata from the analysis of the dipolar traces is available from the repository.‡‡The data associated with this project is avaible online at the following DOIs: Read_me_file: DOI: 10.48420/22172840; LITTER_primary_data: DOI:10.48420/21975953; LITTER_orientation_dependent_analysis: DOI:10.48420/22122566; LITTER_orientation_independent_analysis: DOI:10.48420/22122569; DEER_primary_data: DOI:10.48420/21975932; DEER_orientation_dependent_analysis: DOI:10.48420/22122563.

### Density functional theory calculations

Initial geometries for molecules [1]–[4] were built based on previous reports,^37^ using UCSF Chimera.^42^ Geometry optimizations and spin density calculations were performed *in vacuo* using Gaussian® 16 (revision A.03).^43^ Ground state geometry optimizations of molecules [1]–[4] were carried out in the singlet state, using the PBE1PBE functional and the 6-31g(d) basis set. Geometry optimizations and spin density calculations in the photoexcited triplet state of TPP and ground state doublet of CuTPP were performed using the functional B3LYP, with the basis sets Def2SVP (for H, C, N and O) and DefTZVP (for Cu). Atomic Mulliken spin densities were taken in all cases.

Magnetic tensor orientations (ZFS for TPP triplet, *g* and A(^65^Cu) for CuTPP doublet) were calculated on the optimized geometries of triplet TPP and doublet CuTPP using Orca (release version 4.2.0),^44^ with the functional B3LYP and the basis sets EPR-II (for H, C, N and O) and DefTZVP (for Cu). The RIJCOSX approximation for Coulomb and Hartree–Fock exchange was used, with the auxiliary basis set Def2/J. The spin–spin contribution to the ZFS tensor was calculated using computed UNO (spin-unrestricted natural orbital) determinants,^45^ and no spin–orbit coupling contribution was included. The resulting tensor orientations are in agreement with the literature.^41,46^

### Orientation-dependent simulations

Simulations of libraries of traces corresponding to possible geometrical models of each of the systems were performed using an algorithm based on that reported by Lovett *et al.*,^41^ for DEER. A modified version to take into account the zero-field splitting and triplet populations^34^ was used for the LITTER experiments, and the outline steps for this simulation are provided in the ESI,† Section S4.

In this program the dipolar frequency is calculated using a distributed point dipole model: the dipolar frequency corresponding to each pairwise interaction between pairs of atoms possessing spin density is calculated and combined using a weighting factor proportional to the spin densities on the two atoms in question. For a single molecular conformation and set of experimental conditions the dipolar frequency (*ω*^*n*^_dd_) contribution for each orientation of the molecule with respect to the external magnetic field considered is calculated according to the formula:
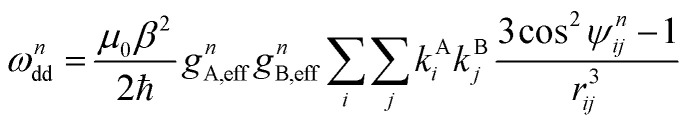
where *g*^*n*^_A,eff_ and *g*^*n*^_B,eff_ are the effective *g*-values at the magnetic field used for the experiment and the probe and pump frequencies respectively, *k*^A^_*i*_ and *k*^B^_*j*_ are the electronic spin densities on the atoms of centers A and B, respectively and *ψ*^*n*^_*ij*_ and *r*_*ij*_ are defined as in Fig. S7 (ESI†). This takes into account the DFT-calculated spin densities for the CuTPP and triplet TPP centres, the values used are provide in the ESI† (Fig. S6 and Tables S2, S3). The complete dipolar spectrum is gained by summing all the different contributions for all orientations of the molecule with respect to the external magnetic field.

The simulation algorithm is based on EasySpin functions to simulate the spectra and resonant fields. As input to this PDS simulation algorithm, the spin system parameters were obtained from fitting trESR or echo-detected field sweep datasets (Fig. S2, ESI† and Fig. 3(a)) using a Matlab® EasySpin routine (pepper function).^39^

Other parameters required for input such as pulse lengths were set to match those used experimentally. The effect of the pump laser is modelled to be equivalent to a microwave pulse with a bandwidth that far exceeds the frequency bandwidth of the trESR spectrum for TPP.

For simplicity, simulations were carried out in the *g*- and ZFS-frames of one of the CuTPP and triplet TPP (Fig. S7, ESI†) centres that was defined at the centre used for detection, for DEER and LITTER, respectively. This required the geometric orientation of the corresponding pump centre to be defined in terms of a set of polar angles (*φ* and *θ*, Fig. S8(a), ESI†), a separation *r* and Euler angles (*α*, *β*, *γ*, Fig. S8(b), ESI†). The initial values for these parameters were determined from the DFT-optimized structures of [1]–[4], provided in the list form *φ*, *θ*, *r*, *α*, *β*, *γ* were 1.45, 0.69, 2.1, 3.33, 1.80, 6.17 for [1]; 1.39, −0.35, 2.4, 5.42, 1.12, 0.04 for [2]; 1.48, 0.65, 2.6, 3.33, 1.79, 6.04 for [3]; 1.05, −0.11, 3.6, 6.25, 0.90, 5.72 for [4]; and 2.29, 0.61, 2.6, 1.23, 2.28, 2.26 for Cu_2_-[3], where all angles are quoted in radians and the separation, r, is given in nm.

From these initial geometries, a library of possible geometric conformers was generated by changing the values of *φ*, *θ*, *r*, in the range Δ*φ* = ±45°, Δ*θ* = ±45° and Δ*r* = ±1 nm (±0.5 nm for [1]) around the initial DFT-optimized structures. For each conformation a random value within the ranges specified above of *φ*, *θ* and *r* was generated. In all conformers the *α*, *β*, *γ* angles were fixed at values corresponding to the optimized orientation of the pump center in the initial DFT model. A total of 2166 structures for [1] and 3971 structures for [2], [3], [4] and Cu_2_-[3] were used to form the libraries for each system.

Dipolar traces were calculated for each structural conformer generated at each field position measured. The libraries of simulated traces were fitted to the experimental datasets using an iterative algorithm similar to that described by Marko *et al.*^47^ until convergence of the root-mean-square deviation (RMSD) was achieved (Fig. S13 and S17, ESI†). Orientation selection effects due to magnetophotselection of the porphyrin by the pump laser (laser 2) were not included in the simulations as this laser pulse was delivered *via* an optical fibre and it was assumed that all polarization was lost within this setup. Orientation selection due to the pump pulse does occur in the DEER simulations due to the limited bandwidth of the pulse. In both experiments orientation selection effects originate from the limited frequency bandwidths of the detection pulses used.

**Fig. 2:**
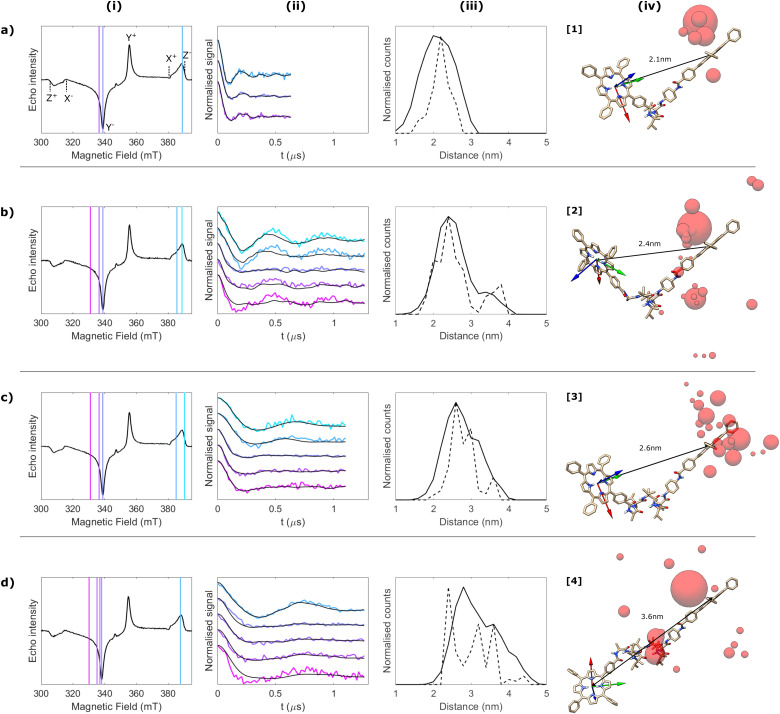
Orientational LITTER study of molecules (a) [1], (b) [2], (c) [3] and (d) [4] at X-band. By columns: (i) echo-detected field-swept ESR spectra of the molecules recorded after 512 nm laser flash. The field positions used to record the LITTER traces are indicated with vertical lines, these positions were selected to sample the orientation information across the spectrum focusing on the *Y* and *Z* transitions. Measurements on the *X* transitions were prevented by low signal intensity at these fields. (ii) Background-corrected and modulation depth-normalized LITTER traces acquired at the different field positions (color) and corresponding orientation-dependent fits (black). The length of traces presented correspond to the length of trace used in the fitting procedure, in some cases longer experimental traces were recorded to enable more accurate background correction, details for this are provided in the data repository for this work.^†^ Modulation depths were ∼10% before normalization in all cases. (iii) Spin–spin distance distributions obtained from the orientation-dependent analysis of the experimental data fitting to a library of simulated traces; the dashed lines indicate the distribution of center-to-center distances (the center-to-center distribution of distances). For comparison, the solid lines represent the distance distribution between each pair of atoms on different chromophores with spin-density that contributes to the distributed point-dipole model used to generate the simulated traces (the point-dipole distribution of distances). (iv) DFT-optimized geometries showing the different positions of the TPP center most contributing to the fits as red spheres, relative to the ZFS-tensor frame of the other TPP triplet (arrows: red = *D*_*x*_, green = *D*_*y*_, blue = *D*_*z*_). The diameter of the spheres is proportional to the number of times a single conformation contributes to the complete fit shown in panels (ii). A rotated view is provided in Fig. S11 (ESI†).

Distance distributions resulting from the orientation-dependent analysis were generated from the molecular geometries contributing to the best fits shown in Fig. 2(ii) and 3(b). We present two types of distance distributions. Firstly, a center-to-center distribution of distances which contains only a single contribution from each conformer corresponding to the center-to-center distance between the two porphyrins. Secondly, we present a point-dipole distribution of distances which also considers the spin–spin distributions, taking into account all of the pairs of atoms possessing spin density considered in the distributed point-dipole mode. Here, each contribution to the distance distribution was weighted by the spin density in the same way as in the calculation of *ω*^*n*^_dd_.

## Results and discussion

This series of bis-labelled peptides [1]–[4] was designed so as to allow for a systematic variation of the distances and relative orientations between the two porphyrin moieties, thus providing a spectroscopic ruler (Fig. 1(b)). They were synthesized as previously reported.^37,38^ The [(α-Me)Val]_*n*_ peptide backbone favors a rigid α-helical conformation, especially in polar solvents such as ethanol, used in this study.^37,38^*In-vacuo* DFT optimizations support the α-helical conformation of the backbones of [2], [3] and [4] and yield porphyrin–porphyrin center-to-center distances of 2.4, 2.6 and 3.6 nm (Fig. 1(c)). The peptide backbone of [1] is too short to form a full α-helical loop and is therefore expected to be more flexible, with a predicted porphyrin–porphyrin center-to-center distance of 2.1 nm according to DFT optimization.

LITTER for all four peptides was carried out at different positions across the ESR spectrum of the photogenerated TPP triplet state at X-band (Fig. 2(i)), in order to obtain orientation-resolved sets of dipolar traces (Fig. 2(ii)). The strong orientational effects, originating from the orientation selection of the narrow-bandwidth microwave pulses in comparison to the large ZFS of the photogenerated TPP triplet state, are clear from the deep dipolar oscillations around *Z*^−^ compared to the very shallow oscillations around *Y*^−^ (the directions of the ZFS-tensor principal axes defining the canonical orientations are indicated in Fig. S7(a), ESI†). It was not possible to acquire LITTER traces at *X*^−^ due to the negligible ESR intensity at this turning point. The significant difference in dipolar frequencies between molecules [2] and [3], despite having very similar porphyrin–porphyrin distances, is another manifestation of the strong dependence of the dipolar interaction on the relative orientation of the dipolar vector and the ZFS tensor of the detection spin center in these experiments.

Orientation-dependent simulations, using a distributed point-dipole model of spin density to calculate the dipolar interactions,^41^ were carried out as described above and similar to our proof-of-concept study.^34^ A library of possible geometries was generated and dipolar traces were calculated for each geometry using parameters matching those used in the experimental traces. Fitting of the experimental LITTER datasets was performed using the library of calculated traces based on an iterative least-squares global fitting procedure,^47^ until convergence of the root-mean-square deviation (RMSD) was achieved (Fig. 2(ii) black, Fig. S13, ESI†). The possible molecular geometries contributing to the library were generated around the DFT-optimized geometries of the molecules, by variation in the position of the dipolar vector relative to the detection center and specified by polar coordinates *φ*, *θ* and *r* (details are given in the experimental section above). The orientation of the pump center D-frame relative to the detection center D-frame, parameterized by Euler angles *α*, *β* and *γ*, is fixed according to the DFT optimized geometry in order to reduce conformational space. Limited molecular motion of the system means that the accessible distributions in the orientations of the two centers will be small. In LITTER the effect of changing the orientation of the pump center is expected to be smaller than in a DEER experiment as the light pump pulse is not orientation dependent. In this way the orientation of the LITTER experiment is much more similar to that of a RIDME experiment, where orientation dependence of the relaxation of the fast-relaxing center is also typically neglected. The electronic spin density delocalization in the photogenerated TPP triplet state was included as calculated by DFT (Fig. S6(a) and Table S2, see ESI† and data repository‡ for full simulation details). Symmetry of the spin density distribution also minimizes effects of small changes in orientation (*α*, *β* and *γ*) of the two centers. The resulting distance distributions were generated from the conformations contributing to the best fit using the distributed point-dipole model, carrying out the weighted sum of pairwise distances between all the atoms bearing significant electronic spin density as calculated by DFT (see Experimental Section for further details, and Fig. S6(a) and Table S2, ESI† for the electronic spin densities used). This distributed point-dipole model intrinsically modifies the dipolar coupling including a distribution in distances and a smaller variation in polar angles (*φ* and *θ*) if the spin distributions are not planar.

This orientation-dependent analysis yielded good fits to the experimental LITTER datasets for all four molecules studied, successfully capturing the orientational effects in the dipolar traces (Fig. 2(ii)). The orientational conformational distributions were plotted as red spheres where the radius is proportional to the contribution of each conformation relative to the DFT-optimized molecular geometries, see Fig. 2(iv) and experimental section for details of DFT calculations. Additionally, distance distributions were plotted, Fig. 2(iii). It is possible to either represent the fitted data as a distribution of center-to-center distances (dashed lines in Fig. 2(iii)), we will refer to this as the center-to-center distribution of distances, or as a distribution of distances used in the point-dipole simulation, between each pair of atoms on different chromophores possessing spin density, while including a weighting based on the spin density associated with that dipole in the distributed point-dipole model (solid lines in Fig. 2(iii)), we will refer to this as the point-dipole distribution of distances. The center-to-center distance calculated from the DFT-optimized molecular geometries is shown in Fig. 2(iv). For molecules [1], [2] and [3], the conformations are in good agreement with the DFT-optimized structure, the center of the second TPP moiety most contributing to the best fits is clustered around the DFT-optimized geometry (Fig. 2(iv)). Additionally, the maximum in the distance distributions is in good agreement with the center-to-center distance predicted by DFT, and the two types of distance distribution are reasonably similar for molecules [1], [2] and [3]. There is a small population of a longer distance (>3 nm) identified in molecules [2] and [3], probably corresponding to a more extended higher-energy conformation of these molecules in frozen solution. Comparing the distance distribution comprised of the center-to-center distances (dashed lines in Fig. 2(iii)), to the single value provided by the static DFT conformation (shown in Fig. 2(iv)), and the distribution of the red spheres representing the fitted conformers (Fig. 2(iv)) shows that there is a spread in the fitted conformers and corresponding distance distribution. This is expected from the fact that these molecules are not completely rigid in solution and therefore many different conformations will be trapped in the frozen state upon flash freezing of the sample in liquid nitrogen.

For molecule [4], in addition to the long distance (∼3.6 nm) corresponding to the extended conformation predicted by DFT, a more intense distance feature centered around 2.8 nm, in the spin–spin point-dipole distribution of distances (Fig. 2(iii) solid line), and 2.2 nm, in the center-to-center distribution of distances (Fig. 2(iii) dashed line), is obtained from the orientation-dependent analysis. Other stable molecular conformations, with porphyrin–porphyrin distances below 3 nm, were identified by *in-vacuo* DFT through bond rotations of the first amino acid of the peptide backbone (Fig. S9, ESI†), suggesting that the short distance feature in the distance distribution could be explained by unwinding of the α-helix to yield alternative conformations. The wider spread of TPP positions compared to molecules [1]–[3] suggests a larger degree of conformational flexibility, consistent with the longer peptide backbone in this molecule. Comparison of the center-to-center distance distribution to the point-dipole distribution of distances for molecule [4] shows a greater variation compared to molecules [1]–[3], the maximum in the center-to-center distribution is lower (2.5 nm) than that in the point-dipole distribution (3 nm). The point-dipole distribution can be thought of as a blurring of the center-to-center distribution taking into account those contributions of the spin-density distribution that are closer and further away than the center-to-center distance for each conformer. In this way the point-dipole distribution may yield a better comparison to the orientational independent analysis for this molecule (Fig. S24, ESI†).

While previous study of these systems used IR spectroscopy to monitor the formation of the stable helical structure by measuring the signal associated with intramolecular N–H hydrogen bonding,^37^ this technique does not report on conformational changes associated with torsional motion of the bonds at the end of the helix where the TPP moieties are linked to the peptide, the rotation of which can cause relatively large changes in the inter-porphyrin distance measured by the LITTER experiment.

**Fig. 3:**
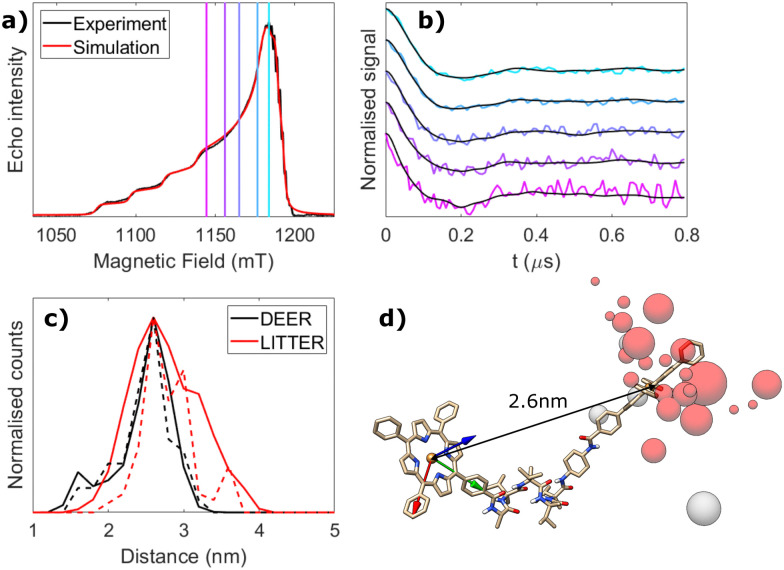
Orientational DEER study of Cu_2_-[3] at Q-band. (a) Echo-detected field-swept ESR spectrum (black) and simulation (red) using the Matlab® EasySpin routine (pepper function), with spin Hamiltonian parameters: *g* = [2.041, 2.051, 2.191], *A*^65^_Cu_ = [72, 89, 639] MHz and *l*_w_ = [2, 2] mT. The field positions used to record the DEER traces are indicated with vertical lines. (b) Background-corrected and modulation depth-normalised DEER traces acquired at the different field positions (colour) and corresponding orientation-dependent fits (black). Modulation depths were ∼2–3% before normalization in all cases. (c) Distance distributions obtained from the orientation-dependent analysis of the DEER (black) and LITTER (red) datasets. In each case the center-to-center distribution of distances are plotted as dashed lines and the point dipole distribution of distance distribution calculated from pairwise distances of atoms possessing spin density on different chromophores is shown as a solid line. (d) DFT-optimised geometry showing the different positions of the CuTPP centre most contributing to the fit as gey spheres, relative to the *g*-tensor frame of the other CuTPP (arrows: red = *g*_*x*_, green = *g*_*y*_, blue = *g*_*z*_). The diameter of the spheres is proportional to the number of times a single conformation contributes to the complete fit shown in panel (b). A rotated view is provided in Fig. S11 (ESI†). The TPP centre positions from Fig. 2(c)-(iv) have been included for comparison (red).

Orientation-selective 4-pulse DEER between Cu(ii) centers was used to benchmark LITTER against a more conventional PDS technique. Cu(ii) was introduced in both porphyrins of [3] to give Cu_2_-[3] and DEER traces were acquired at different positions in the high-intensity region of the CuTPP ESR spectrum, dominated by the in-plane components of the axial *g*-tensor (Fig. 3(a)). Orientation selection in this case originates from the spectral width of CuTPP, due to its large *g*-tensor anisotropy, compared to the narrow bandwidth of the MW pulses used in DEER. Measurements were performed at Q-band to promote increased spin polarization of this Boltzmann-populated spin-half center. However, low ESR signal intensities due to short *T*_m_ (0.78 μs) and the small modulation depths prevented the acquisition of DEER traces in the low-field region of the CuTPP ESR spectrum, where the stronger orientational effects are expected. Therefore, the dataset presented here does not capture the complete orientational dependence of the dipolar interaction and only shows weak orientational effects (Fig. 3(b)) in the region of the spectral maximum. This dataset was acquired over 5 days, as was the corresponding LITTER dataset on [3], showing that the weak spin polarization of the CuTPP center limits the DEER orientational information that can be obtained in a given period of time compared to LITTER with strong spin-polarized photogenerated TPP triplet states. To further quantify the comparison between LITTER and DEER the noise level of the experiment was calculated as the root mean squared deviation of the experimental data from the average value at the end of the trace, where the oscillations have been dampened, and taking the ratio of this with respect to the measured modulation depth to provide a measure of the relative modulation to noise ratio (MNR) of the two techniques (see ESI,† Section S6). The number of scans recorded for each trace was different therefore we also report the MNR values normalized by the square root of the number of scans to provide an MNR value corresponding to a single scan which allows more direct comparison. The MNR values of the DEER and LITTER traces measured are comparable. However, in all cases the normalized MNR value recorded on the signal maximum of the LITTER is about 10–20% higher than that recorded on the signal maximum of the Cu–Cu DEER. It is also important to note that the measured DEER traces are shorter than the measured LITTER traces (1.6 μs for the longest DEER trace and 2 μs for the longest LITTER trace) which will on average improve the MNR of the DEER as less dephasing due to *T*_m_ occurs. Equally, the DEER measurements were recorded at Q-band and the LITTER at X-band. Generally, an enhancement of *ca.* 10-fold has been observed for PDS signals recorded at Q-band compared to those at X-band.^48^ Consequently, while the MNR of the recorded DEER and LITTER traces are the same order of magnitude, if only an X-band spectrometer is available LITTER will provide better quality traces compared to Cu–Cu DEER at X-band, while LITTER is able to provide longer traces with a similar level of MNR to shorter Cu–Cu DEER traces at Q-band.

Orientation-dependent DEER simulations were carried out using the algorithm reported for Cu–Cu DEER,^41^ with the same conformational space used for LITTER on [3], and the same iterative least-squares global fitting procedure was followed.^47^ The resulting distance distribution, generated following a distributed point-dipole model based on the calculated spin density distribution for CuTPP (see Experimental Section for further details, and Fig. S6(b) and Table S3, ESI† for the electronic spin densities used), has its maximum at 2.6 nm, as predicted by DFT and determined by LITTER (Fig. 3(c)). Comparison of the distance and angular dependencies of the conformational distributions fitted to the LITTER and Cu–Cu DEER showed similar distance distributions when the center-to-center distances are considered (Fig. 3(c) dashed traces). However, if we consider a distance distribution formed from all of the points of spin density contributing to the distributed point-dipole model then the distribution for the LITTER data is broader compared with that for the Cu–Cu DEER (Fig. 3(c) solid traces). This is a result of the larger delocalization of spin density for the triplet state compared to copper porphyrins. Looking at the angular distributions (Fig. S18(e) and (f), ESI†), these are broader for the Cu–Cu DEER results suggesting that the Cu–Cu DEER data do not sufficiently constrain the angular dependences. This is further supported as the experimental DEER dataset on Cu_2_-[3] can be simulated reasonably well using the molecular model resulting from the orientation-dependent analysis of the LITTER dataset with [3], as shown in Fig. 2(c) (iv), demonstrating that the two techniques yield results compatible with the same molecular structure (Fig. S18(c), ESI†).

The differences between the conformational distribution fitted to the LITTER traces for [3] and the DEER traces for Cu_2_-[3] can be explained as the DEER dataset does not capture well the relative orientation between *g*_*z*_ and the dipolar vector. This translates into a wider conformational spread, with very few conformations standing out from the rest in the fitting procedure (Fig. 3(d)). In particular, one of the conformations most contributing to the fit, shown in the bottom right corner of Fig. 3(d), is far from the DFT prediction because of a very different orientation of the dipolar vector relative to the *g*-frame of the detection CuTPP (indicated in Fig. 3), despite the spin–spin distance being close to the value predicted by DFT. The limited orientational information contained in the experimental Cu–Cu DEER dataset is a result of the weak signal and low modulation depth that can be achieved on the low field part of the Cu spectrum as demonstrated by simulations of the expected traces in the ESI† (Fig. S19 and S20). The experimentally recoded Cu–Cu DEER traces do not constrain the conformation of the fitting model strongly. By contrast, the TPP spectrum has sufficient signal on the *Z* transitions that LITTER data can be recorded for this orientation with sufficient MNR for analysis. Interestingly, the conformational distribution fitted for the Cu–Cu DEER traces does not fit the LITTER data well, particularly in the region of the *Z* transitions (Fig. S21, ESI†). Consequently, this demonstrates the importance of recording data for this orientation to facilitate an accurate orientation dependent fit. While it may be possible to use an optimized DEER detection scheme with only a small number of field positions to accurately collect orientation independent datasets for bis-copper systems,^40,49^ our results indicate that when orientation dependent analysis is needed a wider range of field positions is required.

In addition, it has previously been shown that even for distances down to 2 nm very similar distributions of dipolar interaction strengths have been recorded in a LiDEER experiment on a porphyrin–nitroxide system and in a DEER experiment on the system intercalated with copper, leading to the extraction of similar spin–spin distance distributions, where detection of orientation selection was limited by experimental bandwidth.^18^ This suggests that the wider electronic spin density delocalization in the photogenerated TPP triplet with respect to CuTPP does not significantly impact the size of the dipolar interactions and it can be attributed to the symmetric electronic spin density distribution in the TPP triplet state. In our comparison, we have already seen that it is important to compare either the center-to-center distance distribution or the distance distribution comprising all of the distances included in a distributed point-dipole model. Comparing the center-to-center distances, the distribution gained from the LITTER data on system [3] is very similar to that found from analyzing the DEER data measured on the Cu_2_-[3] system (dashed lines in Fig. 3(c)). If the distance distributions considering the distributed point-dipole models are compared, that for the LITTER data measured on the free-base porphyrin system is broader than the corresponding one found for the DEER experiment using CuTPP. This can be explained by considering the spin density delocalization that forms the basis of the distributed point-dipole model. If the LITTER traces are analyzed using an orientation independent kernel method, that maps each dipolar frequency to a single distance, or if a distance distribution formed from only center-to-center distances are considered, then the use of two TPP chromophores does not impact the width of the derived distance distribution. However, if the spin density delocalization is included in the distance distribution, for example by using a distributed point-dipole model, then the increased spin density distribution on both centers causes a broadening of the distance distribution. This will have a larger effect on LITTER results compared to LiDEER or LaserIMD, where only one triplet center is used. Importantly, we have shown that the increase in spin density distribution is not significant enough to preclude measurement or analysis of LITTER data measured on free base porphyrins. For a distance of 2.6 nm, corresponding to molecule [3], we have proven by calculation that the inclusion of the spin density distribution has a minimal effect on the dipolar spectra for a range of extreme orientations (Fig. S25 and S26, ESI†). Furthermore, we have successfully measured the inter-porphyrin separation centered at 2.1 nm for molecule [1]. However, at very short distances the use of Cu–Cu DEER may be advantageous due to the smaller spin density delocalization.

The upper limit of the distance measurement for all PDS techniques that do not use Carr–Purcell–Meiboom–Gill methods^50^ is defined by the length of time trace that can be collected, which is limited by the *T*_m_ of the detection spin center. It is therefore expected that LITTER, measured on systems where the detection spin species is a TPP triplet, will have a longer maximum distance than the corresponding DEER using a CuTPP species for detection as *T*_m,TPP-triplet_ > *T*_m,Cu-TPP_, even when the temperature is lowered from 20 to 15 K for the latter (Fig. S3 and S5, ESI†). This provides an advantage for LITTER over Cu–Cu DEER at longer distances. The upper distance limit for the LITTER experiment using a photogenerated TPP triplet for detection will likely be slightly shorter than that measured for the LiDEER TPP triplet–nitroxide systems,^18^ as the modulation depth for the LITTER experiment measured here is smaller than that of LiDEER datasets previously reported and the corresponding signal-to-noise ratio is therefore expected to be lower.^18^

**Fig. 4:**
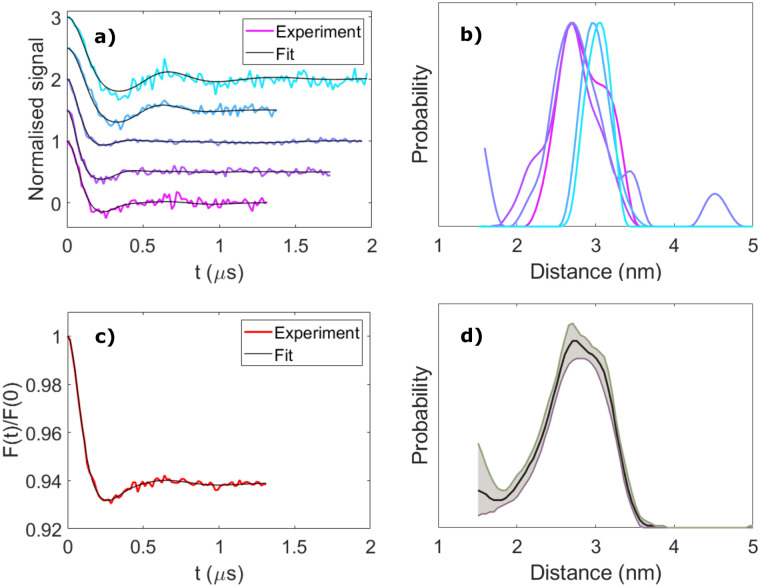
Orientation-independent analysis of LITTER with [3]. (a) Background-corrected and modulation depth-normalised LITTER traces shown in Fig. 2(c)-(ii) (colour) and individual orientation-independent fits by Tikhonov regularization using DeerAnalysis (black), *α* = 10. (b) Spin–spin distance distributions obtained from the analysis shown in panel b. (c) Averaged LITTER trace (red) and corresponding orientation-independent fit by Tikhonov regularization (black). (d) Spin–spin distance distribution obtained from the analysis shown in panel c. 95% confidence bounds (grey) have been estimated using the Comparative Deer Analyzer in DeerAnalysis2022.

To investigate the importance of taking orientational effects into account in LITTER measurements, we have analyzed each individual dipolar trace *via* Tikhonov regularization with the standard DeerAnalysis routine,^51^ which uses an orientation-independent kernel function. The results for molecule [3] are presented in Fig. 4(a) and (b) as an example; the analysis for molecules [1], [2] and [4] can be found in the ESI† (Fig. S22–S24). As expected, significant differences in the shape and position of the distance distribution features calculated with the orientation independent Tikhonov regularization procedure can be observed, indicating that these individual dipolar traces are not representative of the complete distribution when considered individually. For the dipolar traces recorded around the *Z*^−^ region of the TPP triplet ESR spectrum, a distance distribution with a longer average distance is obtained compared to those recorded around *Y*^−^ (Fig. 4(b)). Although all of the individual distance distributions lie within the distribution calculated from the fitting procedure considering the orientation effects and the distributed dipole model, the width of each individual distribution, found by orientation independent analysis of a single trace, is narrower than that found by fitting the complete set of LITTER data considering the orientation selection and the distributed point-dipole model. If only the center-to-center distances are considered in the orientation dependent analysis, the widths of the distributions predicted for the traces recorded close to the *Y* transition become more comparable. This shows that even if the orientational information contained in the dipolar traces is not of interest for the study, measuring a single LITTER trace at a given field position and analyzing it with an orientation-independent method could lead to an erroneous distance distribution, not reflective of the true spin–spin distances, resulting from artefacts due to the orientation dependence of the dipolar interaction.

As optical excitation with non-polarized light is orientation independent, for cases where only the spin–spin distance distribution, and not the orientational distribution, is of interest, an approximate idea of the distance distribution can be obtained by acquiring multiple LITTER traces at different field positions across the triplet spectrum and averaging them, following a similar methodology previously applied to DEER experiments on other systems.^52,53^ In the case of free-base TPP, isomerization of the porphyrin ring nitrogen protonation sites interchanges the *x*- and *y*-axes of the ZFS tensor. The result of this means that reasonable orientation averaging can be achieved by measuring and taking the average, weighted by the spectral intensities at the corresponding field positions, of traces recorded only across the *Z*^−^ and *Y*^−^ transitions, where the signal is most intense, to suppress the orientational effects. We have investigated this by simulating traces corresponding to extreme differences in orientation of two TPP triplet centers where the D-frames are perpendicular and parallel to one another and perpendicular and parallel to the dipolar vector (ESI,† Section S5). In each case, we have compared the trace obtained from a sum of traces calculated across the complete triplet spectrum to that obtained by only summing traces recorded on the *Z* and *Y* transitions (Fig. S25 and S26, ESI†). Results show that the averaged trace is dominated by the *Y* transitions, where the signal intensity is highest. We show, for the extreme orientations, that summing the traces recorded on the *Z* and *Y* transitions provides traces that contain the main frequency contributions as the simulations averaged across the complete spectrum. Furthermore, when we repeated the same procedure for the conformational distribution fitted to the LITTER data for [3], the result from the average of the *Z* and *Y* transitions is even closer to that simulated with complete averaging (Fig. S29 and S30, ESI†). Similar results reproducing the main features in the distance distribution were also seen for system [4], where the data also indicates the presence of a population of conformers with a shorter distance than predicted from the DFT result (Fig. S27 and S28, ESI†).

Correspondingly, we can conclude that for this type of system with two free-base porphyrin chromophores, a distance distribution resulting from Tikhonov regularization analysis of an averaged trace, from data recorded on *Z* and *Y* transitions, can yield reasonable distance distribution information for the complete system that is more accurate than using a single trace. However, detailed interpretation of the shape of this distribution should be avoided as it may still contain orientational artefacts; these will likely be more extreme with more constrained orientations and thus the extent of these differences will depend on the system and the number of traces recorded. The true validity of such an averaging treatment relies heavily on sufficient traces being recorded across different orientations of the ZFS tensor, within the triplet spectrum, to capture all orientations of the dipolar vector with respect to the external magnetic field. For example, it should be noted that only measuring on the *Z* and *Y* transitions cannot be generalized to all types of chromophores as it relies on the symmetry and spectral intensities of the TPP triplet.

The results of this averaging treatment are shown in Fig. 4(c) and (d) for molecule [3] and in Fig. S22–S24 (ESI†) for molecules [1], [2] and [4]. For these systems, based on the number of traces recorded, the distance distributions from the averaged traces are in good agreement with those obtained from the orientation-dependent analysis. Interestingly, for molecule [4], the shorter distance found in the complete orientational dependent LITTER analysis survives the partial orientational averaging and analysis process of the experimental data (Fig. S24(e), ESI†), providing further evidence that it is due to the presence of a second conformation of this molecule in the sample. To validate this, we have used the conformational distribution fitted to the orientationally dependent dataset and simulated a more complete set of traces across the TPP triplet spectrum. These simulated traces have been summed and analyzed using DeerAnalysis with Tikhonov regularization and an orientation independent kernel. The results are presented in the ESI† (Fig. S27 and Fig. S29 for systems [4] and [3], respectively) and again show the continued presence of the shorter distance for system [4].

The combination of these simulation studies, comparison to DFT predictions and orientation-dependent analysis, shows that orientational averaging of LITTER traces capturing the orientational dependence of the dipolar interaction is a valid analysis procedure for these systems to obtain spin–spin distance distributions mostly free of orientational artefacts. It can be used when orientational information is not required, removing the need for the lengthy orientational simulations and iterative fits associated with an orientation-dependent analysis. We have also shown that an accurate spin–spin distance distribution free of orientational artefacts cannot be extracted directly from the orientation-independent analysis of a single LITTER trace, and that the acquisition of multiple dipolar traces at different field positions is necessary even when the orientational information is not of interest, in order to be able to correctly average out the orientational effects.

In this paper we have aimed to present our PDS data in line with the guidelines laid out for publication of DEER data.^54^ However, LITTER and DEER are not the same technique and therefore it is not possible to implement all these guidelines without modification. In the ESI† (Section S7) we present guidelines for publication of LITTER data based on those presented for DEER, indicating where variations occur and why these variations are necessary.

## Conclusion

In conclusion, we have reported a systematic orientational LITTER study of a spectroscopic ruler of bis-porphyrin synthetic model peptides spanning porphyrin–porphyrin distances from 2.1 to 3.6 nm and different relative orientations. Using orientation-dependent simulations and fits, we have obtained information on the conformational distribution of the molecules in frozen solution, in good agreement with the minimum-energy geometries predicted by DFT. This demonstrates that molecules with porphyrin–porphyrin distances as short as 2 nm can be accurately studied with this technique to obtain structural information beyond the distance distribution. By direct comparison to conventional Cu–Cu DEER spectroscopy, we have shown that LITTER is advantageous in allowing data to be recorded in a shorter amount of time and at a higher temperature thanks to the strong spin polarization and longer *T*_m_ of the photogenerated TPP triplet state compared to CuTPP.^18^ In addition, being a single-frequency technique, LITTER removes the restriction of resonator bandwidth that affects DEER experiments, and its orientational resolution does not require the use of high fields as it originates from the highly anisotropic triplet ZFS interaction, unlike for conventional spin-half centers.

This study completes and compliments our previous proof-of-concept LITTER work on a single synthetic model peptide system and sets the base knowledge for the use of this technique in structural investigations of complex biological macromolecules of interest. Other spectroscopic techniques capable of measuring the distance between two chromophores, such as Förster resonance energy transfer (FRET), require the chromophores to adopt different orientations relative to the polarization of the light, and are therefore limited by the particular structure of the systems studied.^55^ LITTER, on the other hand, is free of this limitation and can directly measure the relative orientation of the chromophores, information that cannot easily be obtained from all FRET experiments. We envision that the structure of macromolecular systems containing endogenous photoexcitable moieties, such as light-harvesting proteins and flavoproteins, and systems modified with several photoswitchable triplet labels, could be studied with LITTER. This removes the need for any spin-labeling with nitroxide radicals, which can cause structural distortions, leading to the possibility of more accurate PDS measurements in these systems. Furthermore, nitroxide radicals are known to be unstable in the intracellular environment,^35,36^ limiting the current applications of PDS methods in near-native environments, such as in cells. As photogenerated triplets can be generated within cells,^56^ LITTER is well suited for in-cell structural studies and could be combined with optical microscopy techniques to correlate protein structural information to its location inside the cell.

## Conflicts of interest

There are no conflicts to declare.

## Supplementary Material

CP-026-D3CP03454B-s001

CP-026-D3CP03454B-s002
