# Supplementary material for: Determining and controlling conformational information from orientationally selective light-induced triplet–triplet electron resonance spectroscopy for a set of bis-porphyrin rulers
Source: Phys Chem Chem Phys. 2024 Jan 3;26(3):2589–602. doi: 10.1039/d3cp03454b (PMC10793979; doi:10.1039/d3cp03454b)
Supplement: CP-026-D3CP03454B-s001 [file CP-026-D3CP03454B-s001.pdf]

Electronic Supplementary Material (ESI) for Physical Chemistry Chemical Physics.
This journal is © the Owner Societies 2023

# Supporting Information for Determining and controlling conformational information from orientationally selective Light-Induced Triplet–Triplet Electron Resonance spectroscopy for a set of bis-porphyrin rulers

*Arnau Bertran,*[a] Marta De Zotti,[b,c] Christiane R. Timmel,[a] Marilena Di Valentin* [b,c] and Alice M. Bowen*[d]*

a Arnau Bertran and Prof. Christiane R. Timmel, Centre for Advanced Electron Spin Resonance and Inorganic Chemistry Laboratory, Department of Chemistry, University of Oxford, South Parks Road, Oxford OX1 3QR, United Kingdom, arnau.bertran@chem.ox.ac.uk

b Prof. Marta De Zotti and Prof. Marilena Di Valentin, Department of Chemical Sciences, University of Padova, Via Marzolo 1, 35131 Padova, Italy

c Prof. Marta De Zotti and Prof. Marilena Di Valentin
Centro Interdipartimentale di Ricerca "Centro Studi di Economia e Tecnica dell'energia Giorgio Levi Cases"
35131 Padova, Italy, marilena.divalentin@unipd.it

d Dr Alice M. Bowen, The National Research Facility for Electron Paramagnetic Resonance, Department of Chemistry and Photon Science Institute, The University of Manchester, Oxford Road, Manchester M13 9PL, United Kingdom, alice.bowen@manchester.ac.uk

CONTENTS

S1. Spectroscopic characterization of [**3**]

S2. Spectroscopic characterization of $Cu_2$-[**3**]

S3. Computational results

S4. Orientation-dependent analysis

S5. Orientation-independent analysis

S6. Modulation depth to noise ratio

S7. Reporting checklist and data deposition checklist for LITTER

## S3. RESULTS

### S1. Spectroscopic characterization of [3]

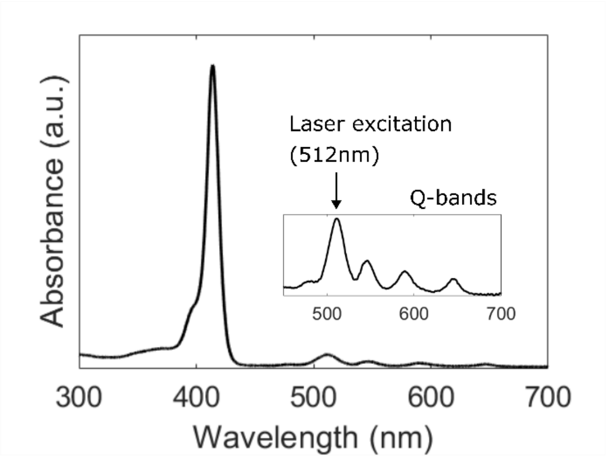


**Figure S1.** Room-temperature UV-Vis absorption spectrum of [**3**] in $d_6$-ethanol. (inset) Zoom in the porphyrin Q-bands, indicating the laser excitation wavelength (512 nm) used for the light-induced experiments.

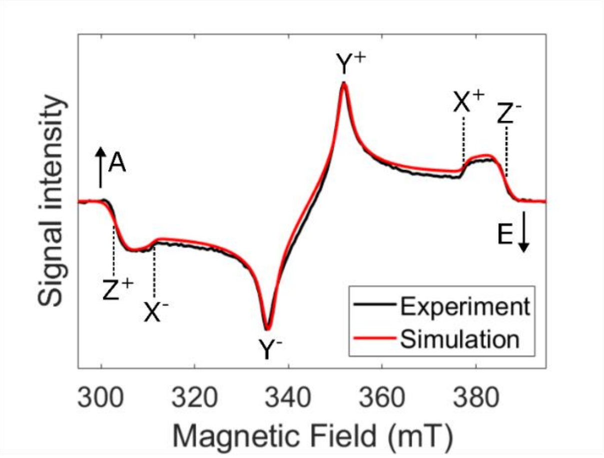


**Figure S2.** trESR spectrum of [**3**] at 20 K measured after laser flash (black) and simulation (red) using the Matlab® *EasySpin* routine (pepper function)[1] with the following spin Hamiltonian parameters: D = 1165 MHz, E = -235 MHz, D-strain = 60 MHz, E-strain = 0 MHz, g = 2.007, lw = 0.7 mT and triplet state sublevel populations $p_x$ = 0.32, $p_y$ = 0.46 and $p_z$ = 0.23. The turning points, corresponding to the canonical orientations of the anisotropic ZFS tensor, are labelled ($Z^+$, $X^-$, $Y^-$, $Y^+$, $X^+$ and $Z^-$, from left to right) and the absorptive (A) and emissive (E) parts of the spectrum are indicated.

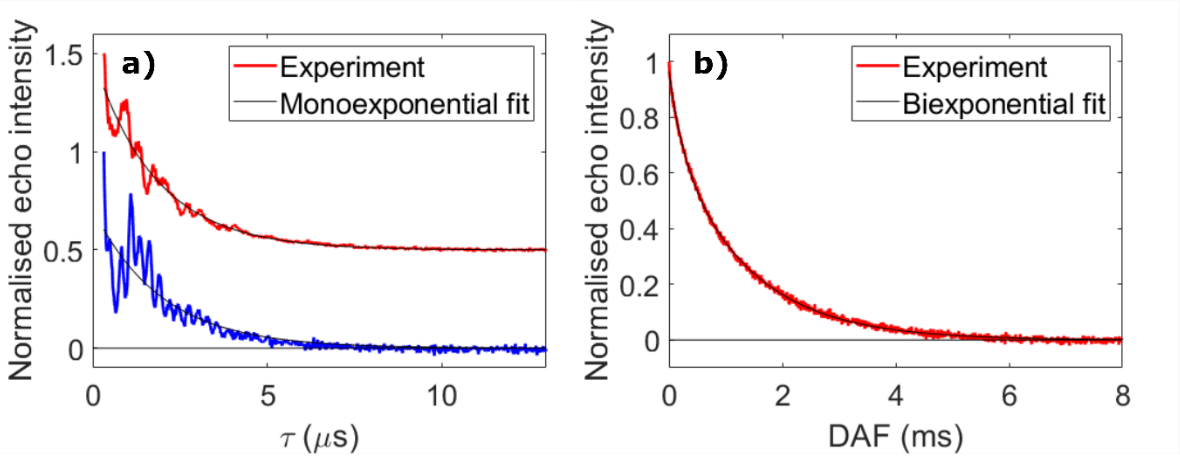


**Figure S3.** Relaxation experiments with [**3**] at 20 K. (a) Phase-memory experiments at the turning points $Y^-$ (red) and $Z^-$ (blue), and corresponding monoexponential fits (black) with $T_m$ values of (1.80 ± 0.04) μs and (2.1 ± 0.1) μs, respectively. The experiments measured at the other positions further downfield showed the same ESEEM features as $Y^-$. (b) Delay-after-flash (DAF) experiment at $Y^-$ (red) and corresponding biexponential fit (black) with lifetimes of (0.23 ± 0.01) ms and (1.32 ± 0.01) ms.

**Table S1.** Phase-memory times for the different free-base molecules at 20 K, measured at the $Y^-$ and $Z^-$ turning points of the porphyrin triplet spectrum.

| Molecule | $T_m$ (μs) | |
|---|---|---|
| | @ $Y^-$ | @ $Z^-$ |
| **[1]** | 1.85 ± 0.04 | 2.1 ± 0.1 |
| **[2]** | 1.56 ± 0.04 | 1.78 ± 0.09 |
| **[3]** | 1.80 ± 0.04 | 2.1 ± 0.1 |
| **[4]** | 1.32 ± 0.03 | 1.48 ± 0.09 |

## S2. Spectroscopic characterization of $Cu_2$-[3]

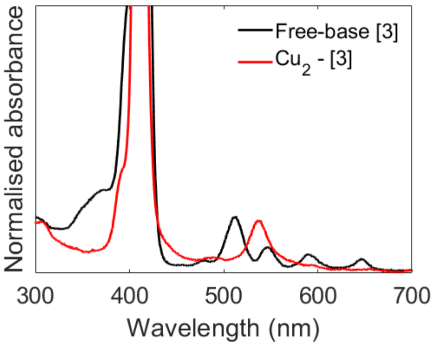


**Figure S4.** Room-temperature UV-Vis absorption spectrum of [**3**] before (black) and after (red) Cu(II) complexation, in $d_6$-ethanol. Absorbances have been normalized to the absolute maximum (Soret band).

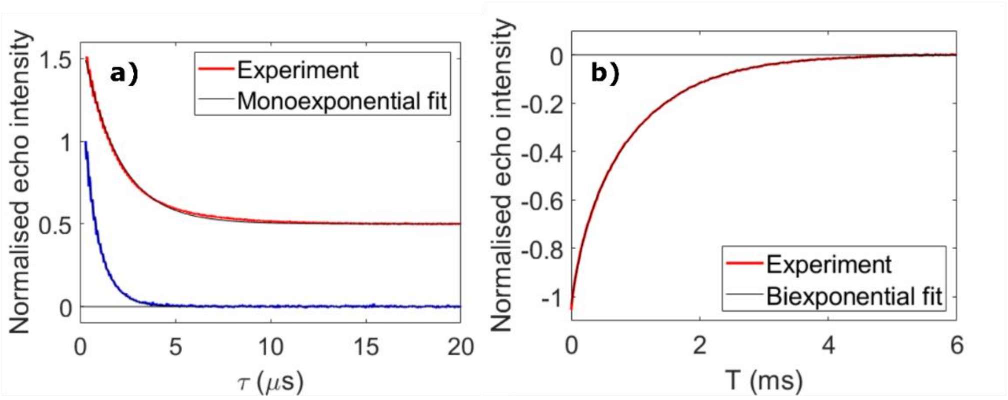


**Figure S5.** Relaxation experiments with $Cu_2$-[**3**] at Q-band and 15 K. (a) Phase-memory experiments at the lowest (blue) and highest (red) field positions used for DEER, and corresponding monoexponential fits (black) with $T_m$ values of (0.78 ± 0.01) µs and (1.89 ± 0.02) µs, respectively. (b) Inversion-recovery experiment at the spectral maximum (red) and corresponding biexponential fit (black) with lifetimes of (0.19 ± 0.01) ms and (1.02 ± 0.05) ms.

## S3. Computational results

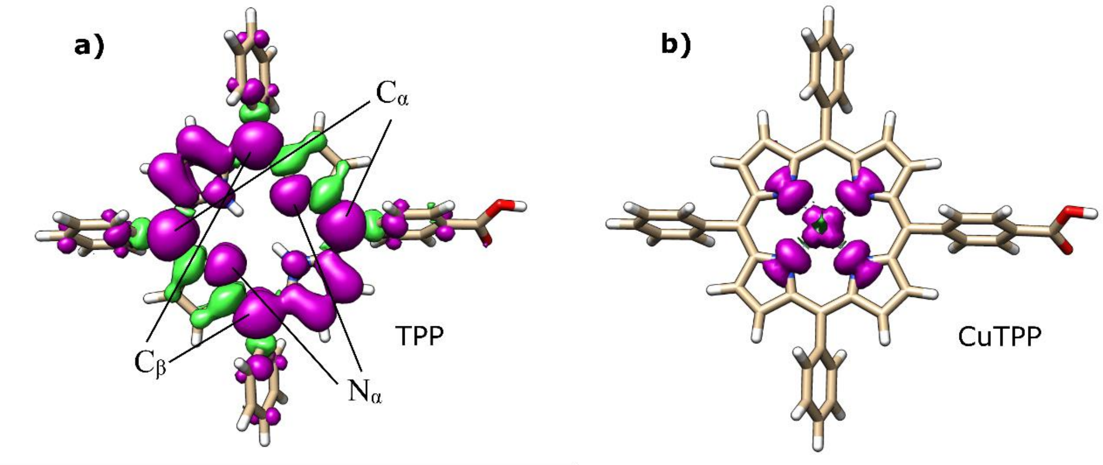


**Figure S6.** Electronic spin density in the triplet state of TPP (a) and in the doublet state of CuTPP (b), calculated using Gaussian 16, functional B3LYP, basis sets Def2SVP and DefTZVP (for Cu). The Mulliken atomic spin density values for the atoms indicated in (a) and for (b) are given in Tables S2 and S3, respectively.

**Table S2.** TPP triplet electronic spin density values (taken as Mulliken atomic spin densities) used for the orientation-dependent LITTER simulations and to generate the LITTER spin–spin distance distributions.

| Atom | Spin density |
|---|---|
| $N_\alpha$ | 0.27 |
| $C_\alpha$ | 0.36 |
| $C_\beta$ | 0.34 |

**Table S3.** CuTPP electronic spin density values (taken as Mulliken atomic spin densities) used for the orientation-dependent DEER simulations and to generate the DEER spin–spin distance distributions.

| Atom | Spin density |
|---|---|
| Cu | 0.58 |
| N | 0.10 |

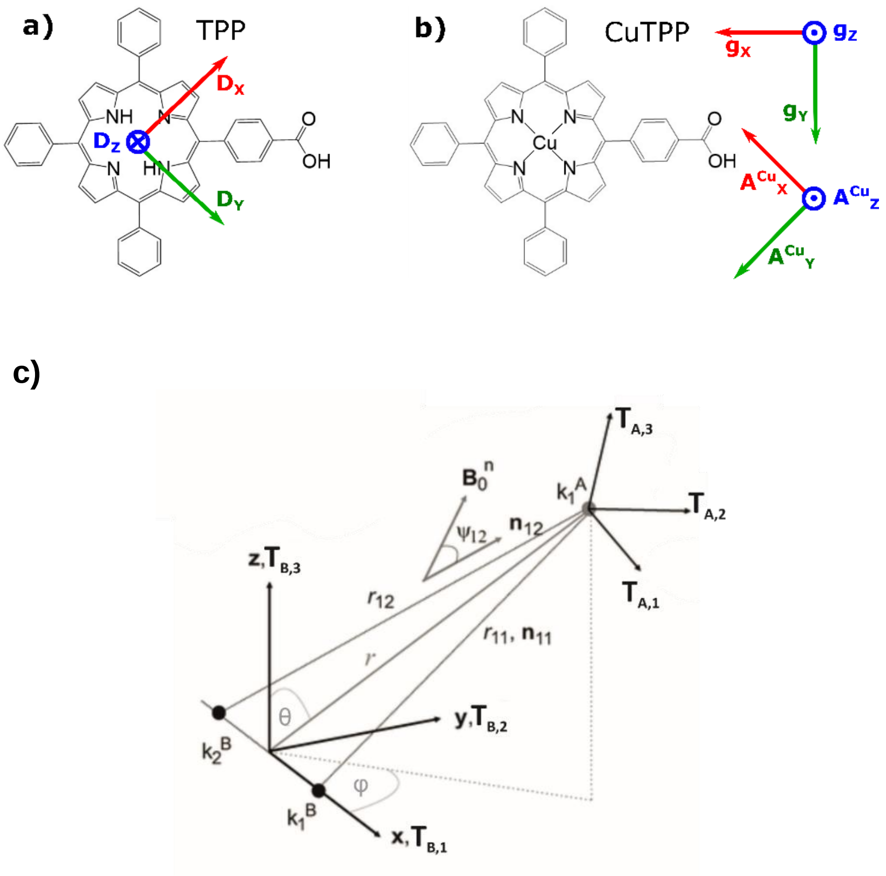


**Figure S7.** Magnetic tensor principal axes for (a) the photoexcited triplet state of TPP (ZFS) and (b) the ground state doublet of CuTPP (g and $A^{65}{}_{Cu}$), calculated using Orca, functional B3LYP, basis sets EPR-II and DefTZVP (for Cu). (c) Geometric model for the orientation-dependent simulation of Pulsed Dipolar Spectroscopy traces between two weakly-coupled spin centres A, B with anisotropic g- or ZFS-tensors $g_A$, $g_B$ or $D_A$, $D_B$, these are denoted $T_A$ and $T_B$ in the figure, and the axes labelled 1, 2, 3. The polar angles θ and φand the separation r defining the relative position of the two centers are shown in grey and the relative orientation of the two centers is defined by Euler angles α, β, γ following a zyz passive convention (not shown). $\Psi_{12}$ is the angle between the orientation of $B_0^n$ shown and the unit vector $n_{12}$ between the point denoted $k_1^A$ and $k_2^B$ on centers B and A, a similar angle can be calculated between unit vector $n_{11}$ and $B_0{}^n$.

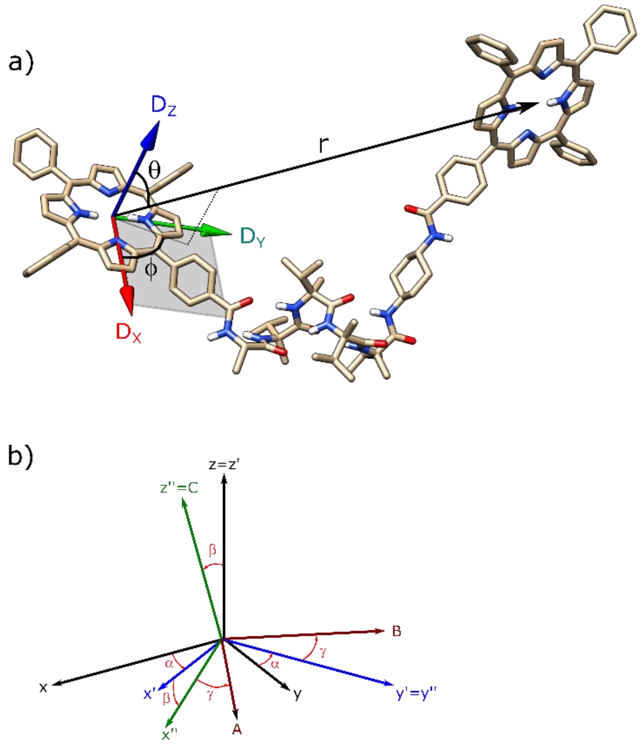


**Figure S8.** Angle definitions for the orientation-dependent simulation of pulsed dipolar spectroscopy traces between two porphyrin triplet states. (a) Polar angles θ and φ and the separation r defining the position of the pump triplet (right) relative to the detection triplet (left). (b) Euler angles α, β, γ defining the relative orientation between the ZFS tensor frames of the two triplet states, following a zyz convention to rotate the ZFS tensor frame of the detection triplet (xyz, black) into the ZFS tensor frame of the pump triplet (ABC).

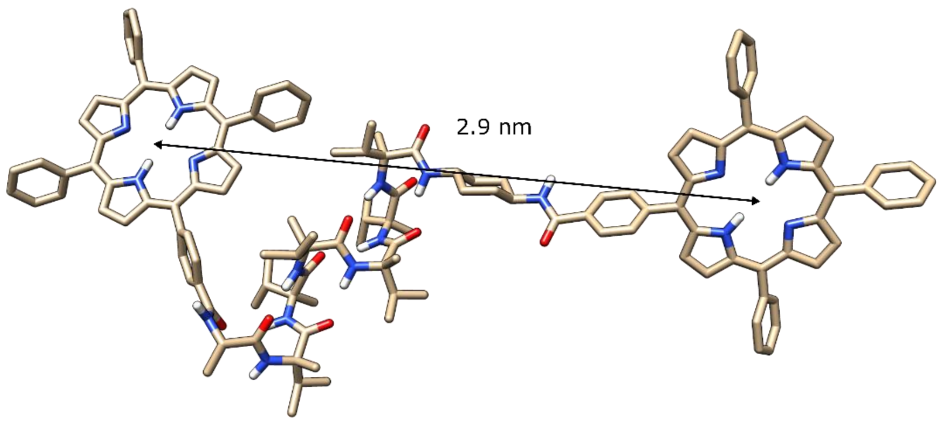


**Figure S9.** Higher local energy minimum conformation of [**4**] (+7 kJ/mol) identified by *in-vacuo* DFT through the unwinding of the first amino acid of the α-helix at the N-terminus, leading to a shorter porphyrin–porphyrin distance of 2.9 nm.

## S4. Orientation-dependent analysis

The main steps of the orientation simulation algorithm for the calculation of a pulsed dipolar spectroscopy trace at a given $B_0$ field value for a single geometric conformation are enumerated below:

1) Define the unit vectors $\{n_{ij}\}$ and spin-spin distances $\{r_{ij}\}$ for all the pairwise dipolar interactions in the model of the spin system.

2) Generate a grid of n magnetic field orientations $\{B_0^n\}$, making angles $\Psi_{ij}^n$ with respect to the pairwise dipolar vectors $\{n_{ij}\}$, and calculate the solidangle weights $w^n$ for each $B_0^n$ vector.

3) Determine the subset of $T_A$ orientations, where T can be either a g-tensor for a DEER experiment of a ZFS-tensor for the LITTER experiment, excited by the detection pulses at a given $B_0^n$ orientation ($f_A^n$), using the simulated ESR spectrum of centre A ($I_A^n$) and the calculated excitation profile of the detection pulses ($P_{det}$).

$$f_A^n = \int_0^\infty I_A^n(\Delta\omega_A)P_{det}(\Delta\omega_A)\mathrm{d}\omega$$

4) Determine the $T_B$ orientations corresponding to excited $T_A$ orientations, using the structural model defined by spherical polar coordinates (φ, θ, r) and Euler angles (α, β, γ).

5) Determine the subset of these $T_B$ orientations that is also excited by the pump pulse ($c_{mod}^n$), using the simulated ESR spectrum of centre B ($I_B^n$ )and the calculated excitation profile of the pump pulse ($P_{pump}$).

$$c_{mod}^{n} = \int_0^{\infty} I_B^n(\Delta\omega_B) P_{pump}(\Delta\omega_B) \mathrm{d}\omega$$

This fraction of flipped B-spins determines the contribution of orientation n to the modulation depth. In the case of LITTER $c_{mod}^{n} = 1$ for all orientations as excitation of the pump center B is assumed to be orientation independent.

6) Calculate the strength of the dipolar interaction for each pair of excited A-B orientations by summing over all the A-B pairs of atoms bearing electronic spin density. The dipolar frequency for the n-th pairwise component is given by:

$$\omega_{dd}^{n} = \frac{\mu_0 \beta^2}{2\hbar} g_{A,eff}^{n} g_{B,eff}^{n} \sum_i \sum_j k_i^A k_j^B \frac{3\cos^2\psi_{ij}^n - 1}{r_{ij}^3}$$

where $g_{A,eff}^{n}$ and $g_{B,eff}^{n}$ are the effective g-values at the magnetic field used for the experiment and the probe and pump frequencies respectively, $k_i^A$ and $k_j^B$ are the electronic spin densities on the atoms of centers A and B, respectively and $\psi_{ij}^n$ and $r_{ij}$ are defined as in Figure S7.

7) Construct the contribution of orientation $B_0^n$ to the dipolar spectrum. If $c^{n}_{mod} = 0$ then a zero-frequency component is added to the spectrum with weight $w^n f_A^n$. If $c^{n}_{mod} > 0$, then $\omega_{dd}^{n}$ is calculated and contributions of $\pm\omega_{dd}^{n}$ weighted by ½ $c^{n}_{mod} w^n f_A^n$. added to the modulated part of the dipolar spectrum and a zero-frequency component weighted by $(1-c^{n}_{mod})$ $w^n f_A^n$. added to the non-modulated part of the spectrum.

8) Once all orientations of Sum the contributions over all orientations $B_0^n$ have been considered, the time trace can be calculated from the frequency domain by a Fourier transform.

The above procedure can be repeated for experiments measured at different $B_0$ field values or pump-probe offsets.

If an ensemble of structural models is generated, for instance, by small geometry distortions from a given structure to emulate the flexibility of spin systems in real ESR samples, a library of orientation-dependent DEER traces for this model can be simulated. An iterative minimum-squares fitting procedure can be then followed to determine the linear combination of simulated traces that provide the best description of the experimental data. The coefficients of this best-fitting linear combination of traces inform on the relative populations of the model structures in the sample and can be used to refine the initial model. Such a fitting procedure follows these steps:

1) Select, from all the traces of the library, the one that gives the minimum root-mean-square deviation (RMSD) from the experimental data and store it.

2) Find, from all the traces of the library, the one that, when added to the stored trace, gives the minimum RMSD from the experimental data.

3) Replace the stored trace by this new minimum-RMSD combination of traces.

4) Repeat 2-3 until a threshold RMSD or a maximum number of iterations is reached. The frequency of occurrence of each simulated trace in the final minimum-RMSD combination, normalised to the total number of iterations performed, gives the relative weight of the corresponding structure contributing to the refined model.

Additional results from the orientation dependent analysis are presented in the figures below, descriptions are provided in the figure captions.

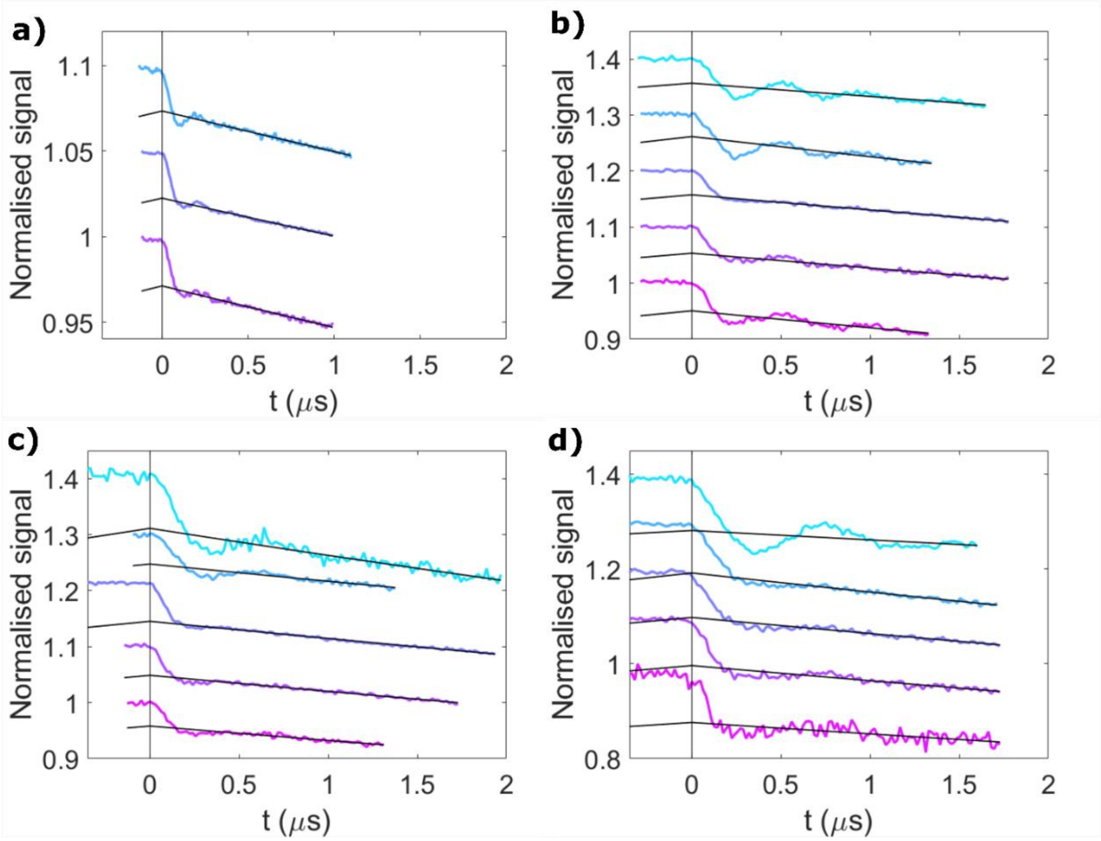


**Figure S10.** Raw LITTER traces for molecules (a) [**1**], (b) [**2**], (c) [**3**] and (d) [**4**] (colour), and three-dimensional homogeneous backgrounds fitted in DeerAnalysis2022 (black).

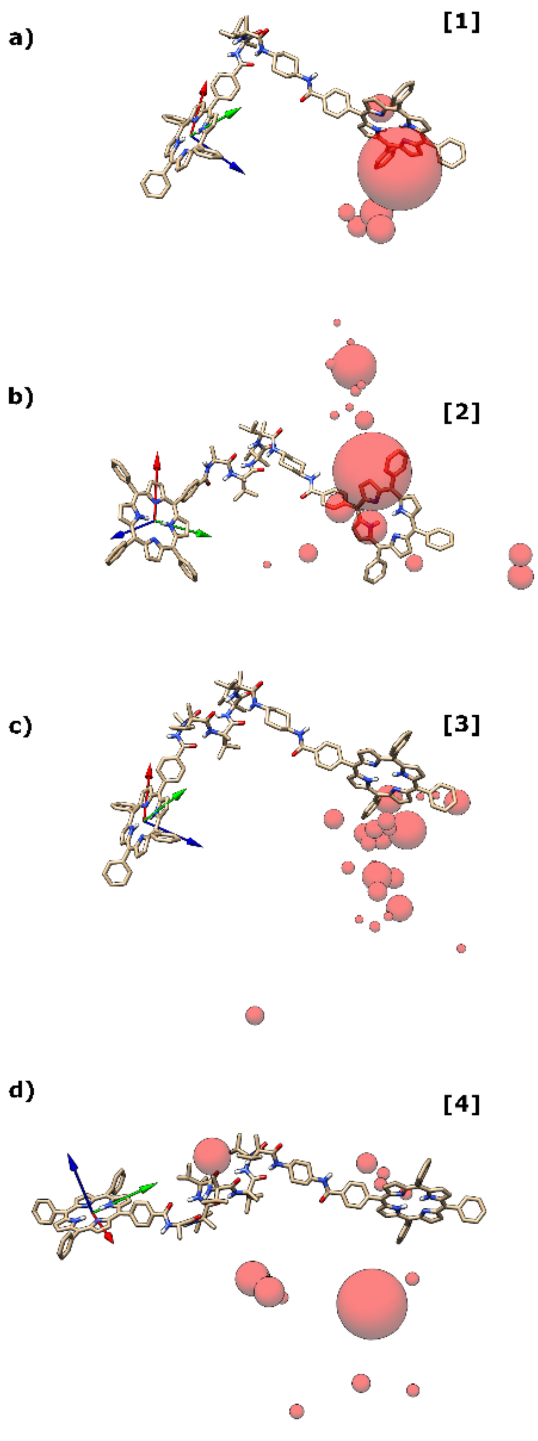


**Figure S11.** Rotated views of Figure 2 a-d panels iv.

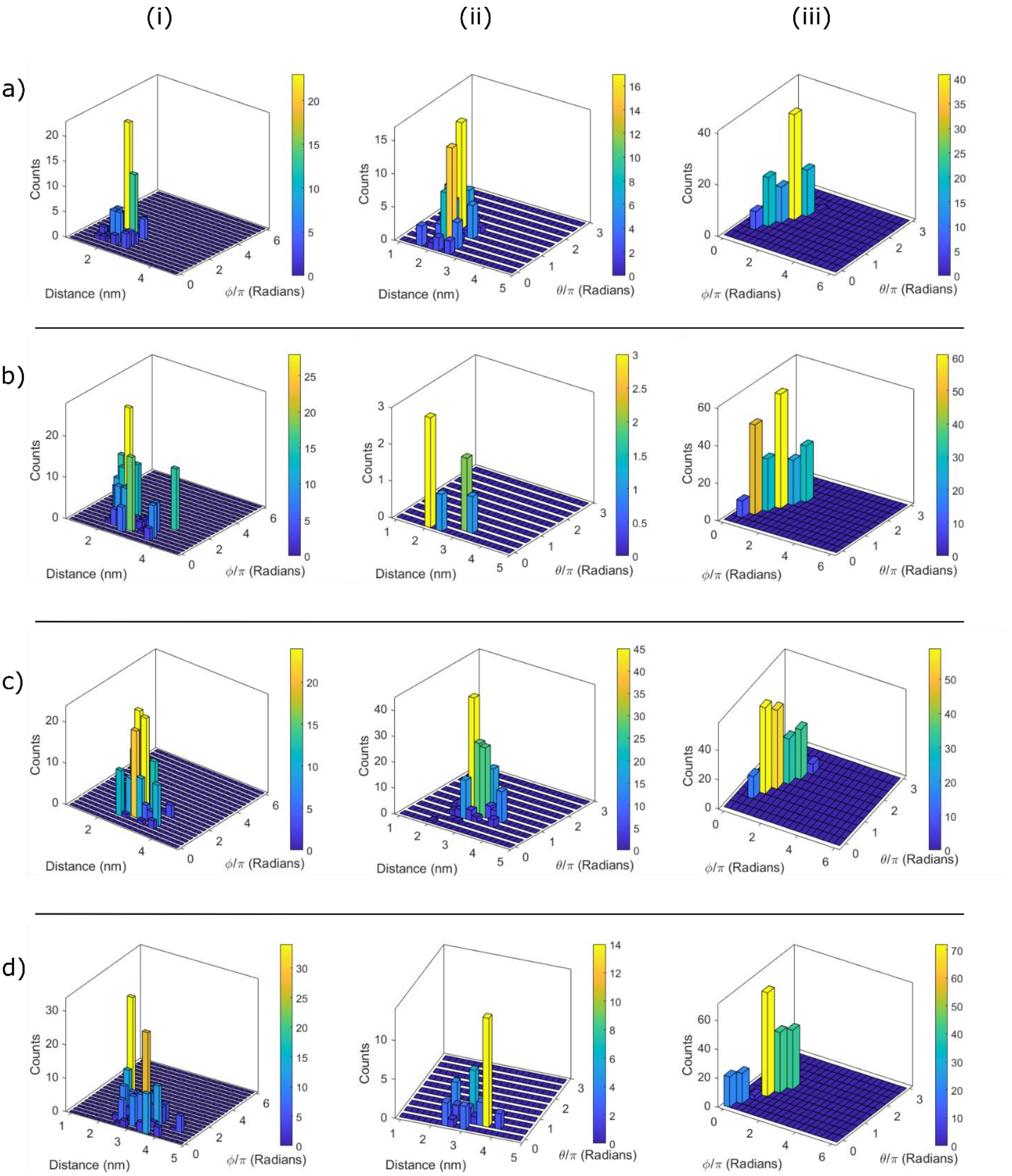


**Figure S12.** Distance-angle and angle-angle histograms for the orientational LITTER fits of molecules (a) [**1**], (b) [**2**], (c) [**3**] and (d) [**4**] shown in Figure 2. (i) Distance *vs* φ, (ii) distance *vs* θ and (iii) φ *vs* θ. The three parameters are defined according to Figure S8a.

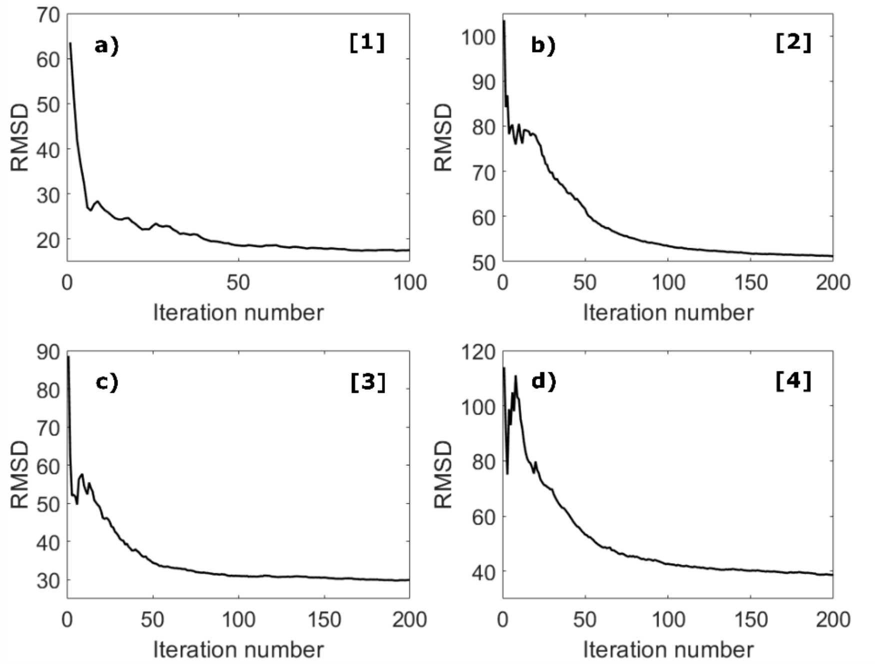


**Figure S13.** Root-mean-square deviation plots for the orientational LITTER fits of molecules (a) [**1**], (b) [**2**], (c) [**3**] and (d) [**4**].

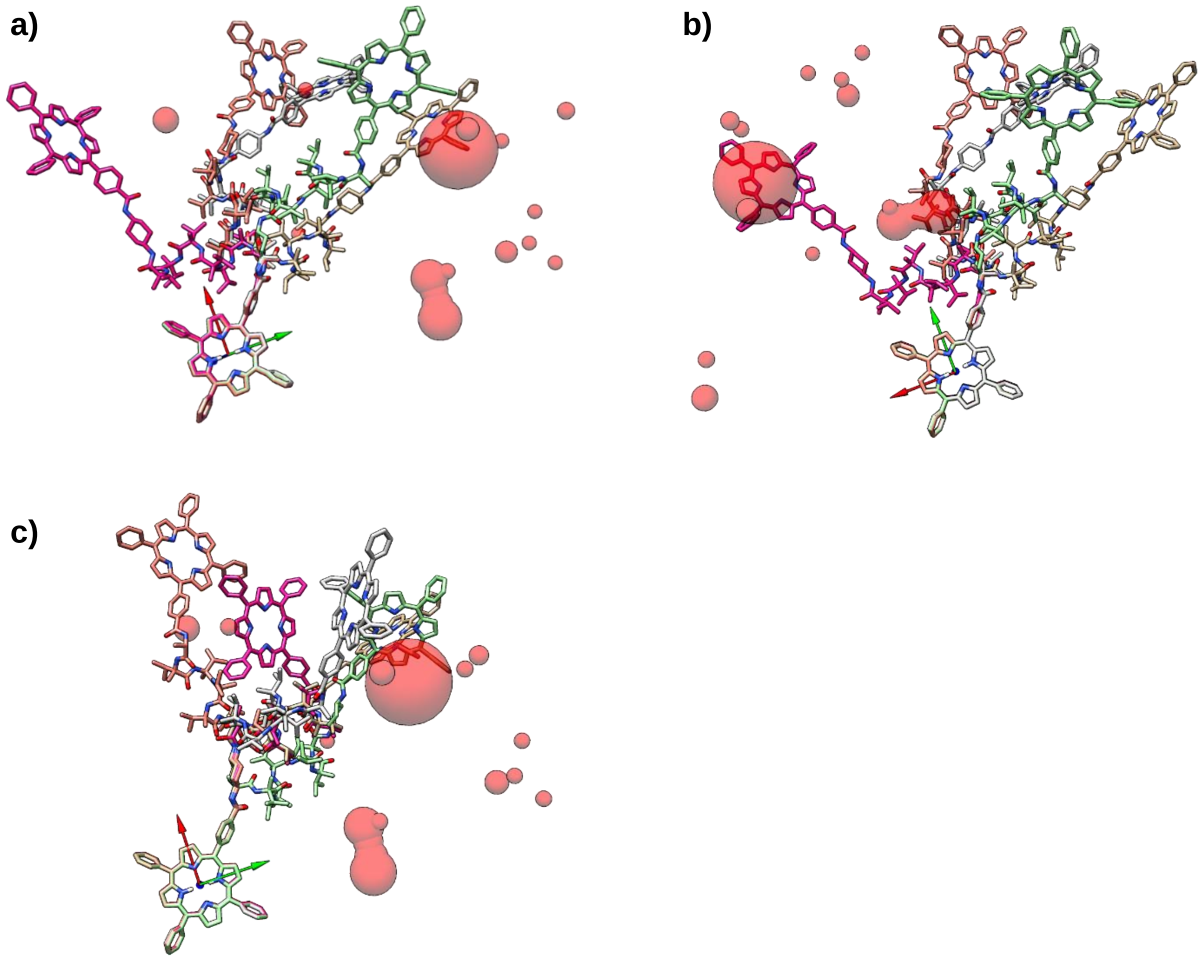

**Figure S14.** Orientational LITTER fit results for molecule [**4**] overlayed with the other higher-energy local minima conformers of [**4**] identified by DFT geometry optimization. The pink structure corresponds to the conformer shown in Figure S9. This shows the flexibility of structures that might be expected in the ensemble and how these can explain the broad distribution of distances and orientations fitted to this dataset. a) The orientation is as shown in Figure 4d panel iv (brown structure and red spheres) and structures are displayed with the same aligned ZFS tensor frames of the N-terminal porphyrin triplet state. b) Due to the tautomerization of the free-base porphyrin it is also possible to rotate the ZFS tensor by 90°, this view is presented here. c) It is not possible to say which porphyrin center in a molecule is the detection center and which the pump center, in this figure the ZFS of the other porphyrin is aligned.

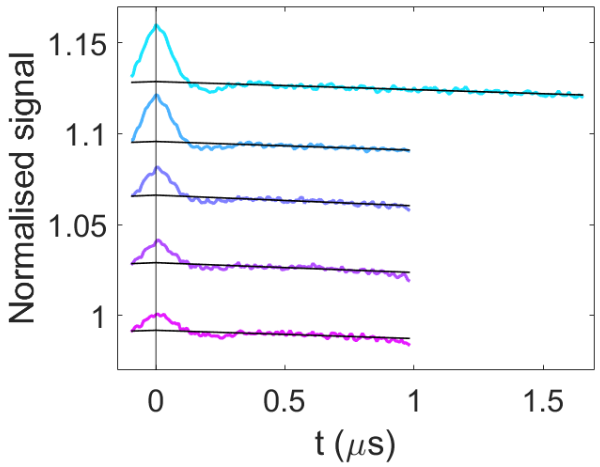


**Figure S15.** Raw DEER traces for $Cu_2$-[**3**] (colour) and three-dimensional homogeneous backgrounds fitted in DeerAnalysis2022 (black).

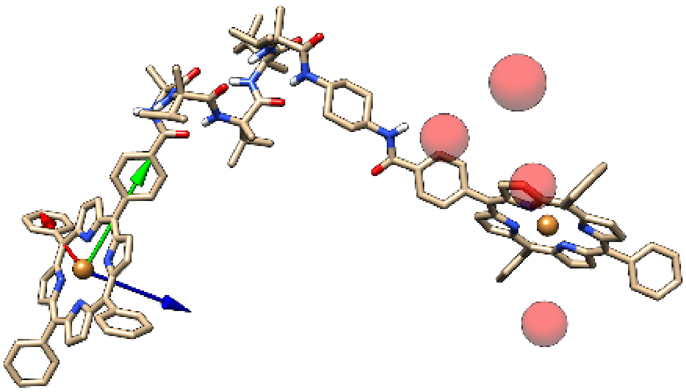

**Figure S16.** Rotated view of Figure 3 d.

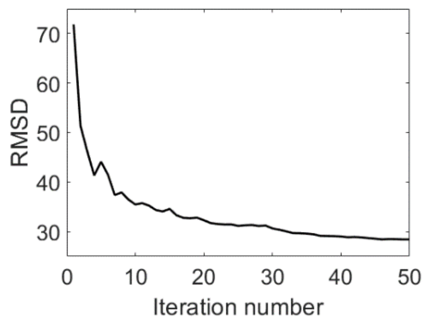


**Figure S17.** Root-mean-square deviation plots for the orientational DEER fit of $Cu_2$-[**3**].

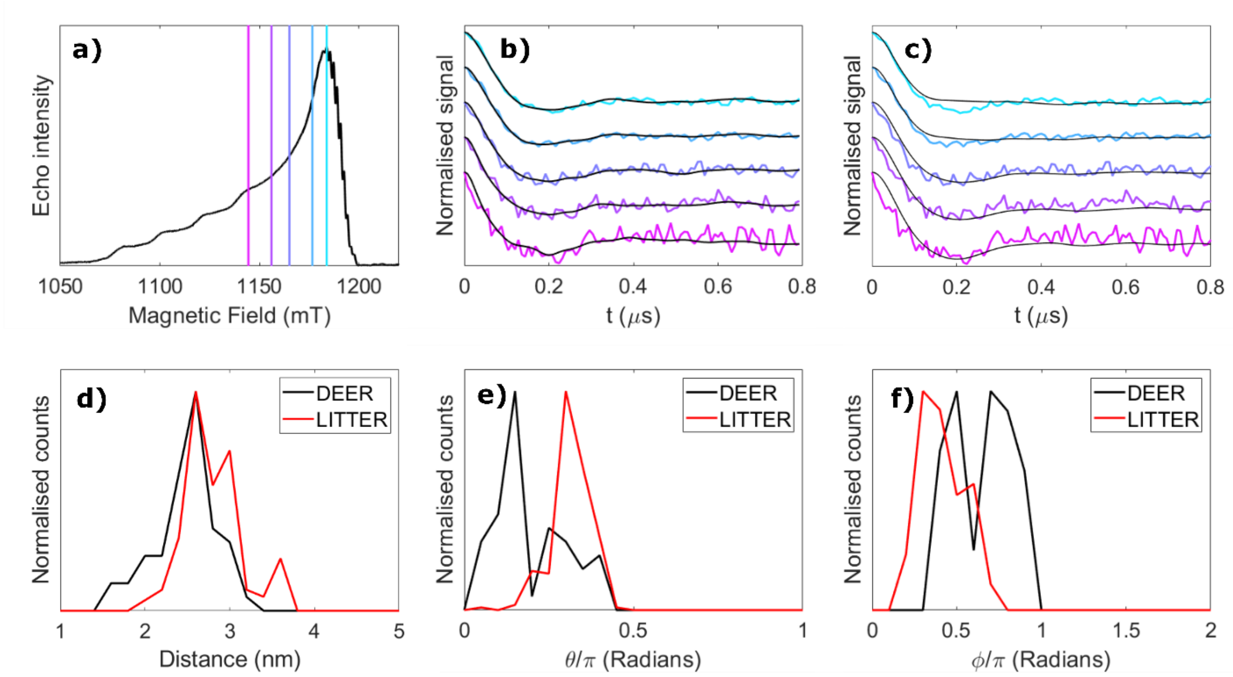


**Figure S18.** Comparison between (b) the results of the orientation-dependent fit to the DEER dataset with $Cu_2$-[**3**] shown in Fig. 3, and (c) the simulation of the Cu–Cu DEER traces using the molecular model resulting from the orientation-dependent analysis of the LITTER dataset with [**3**] shown in Fig. 2 (c). Comparison of the distribution in center-to-center distances (d), and polar angles θ (e) and φ (f) fitted for the DEER and LITTER conformational distributions used to simulate the traces in (b) and (c) respectively.

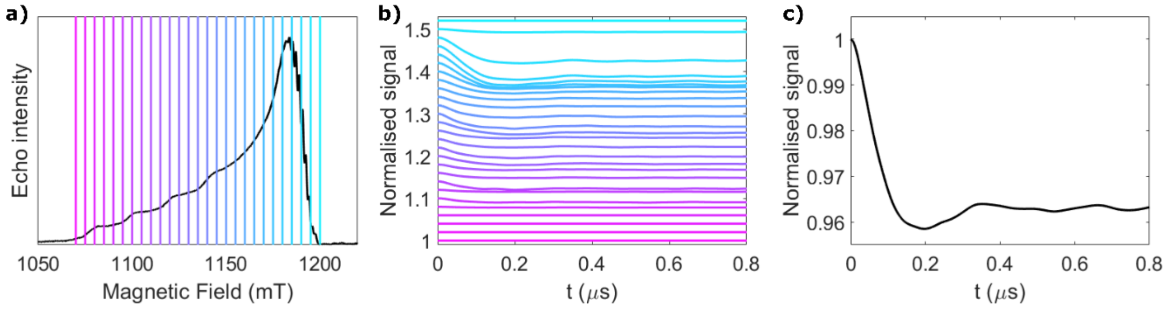


**Figure S19.** (a) Echo-detected field-swept spectrum of $Cu_2$-[**3**] and fields used for the simulation. (b) Simulated DEER traces for $Cu_2$-[**3**] using the molecular model obtained from the fit to the DEER dataset of $Cu_2$-[**3**] (Fig. 3). (c) Orientationally averaged DEER trace obtained by summing traces in b weighted by the spectral intensities in a.

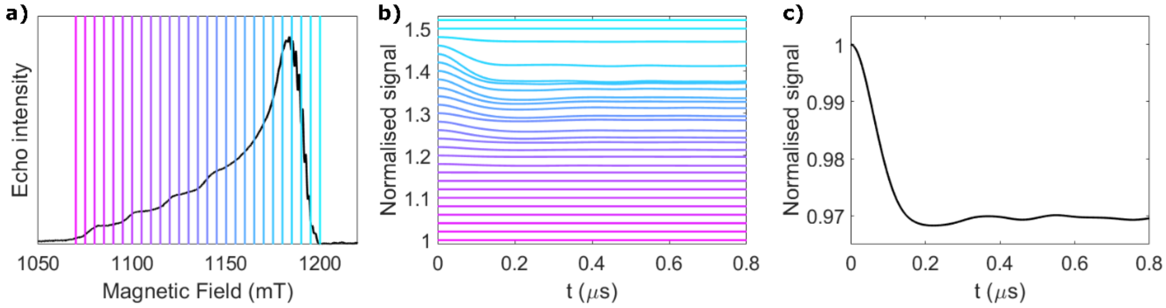


**Figure S20.** (a) Echo-detected field-swept spectrum of $Cu_2$-[**3**] and fields used for the simulation. (b) Simulated DEER traces for $Cu_2$-[**3**] using the molecular model obtained from the fit to the LITTER dataset of [**3**] (Fig. 2 c). (c) Orientationally averaged DEER trace obtained by summing traces in b weighted by the spectral intensities in a.

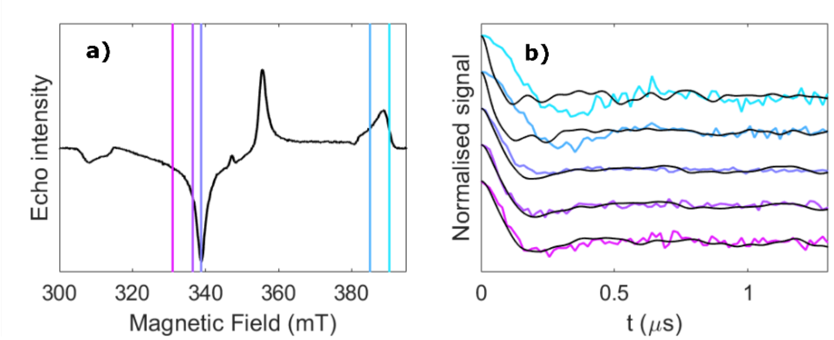


**Figure S21.** (a) Echo-detected field-swept spectrum of [**3**] after photoexcitation and fields used for the LITTER experiments and simulations. (b) Experimental (colours) and simulated LITTER traces (black) using the molecular model obtained from the fit to the DEER dataset of $Cu_2$-[**3**] (Fig. 3). Modulation depths are normalised.

## S5. Orientation-independent analysis

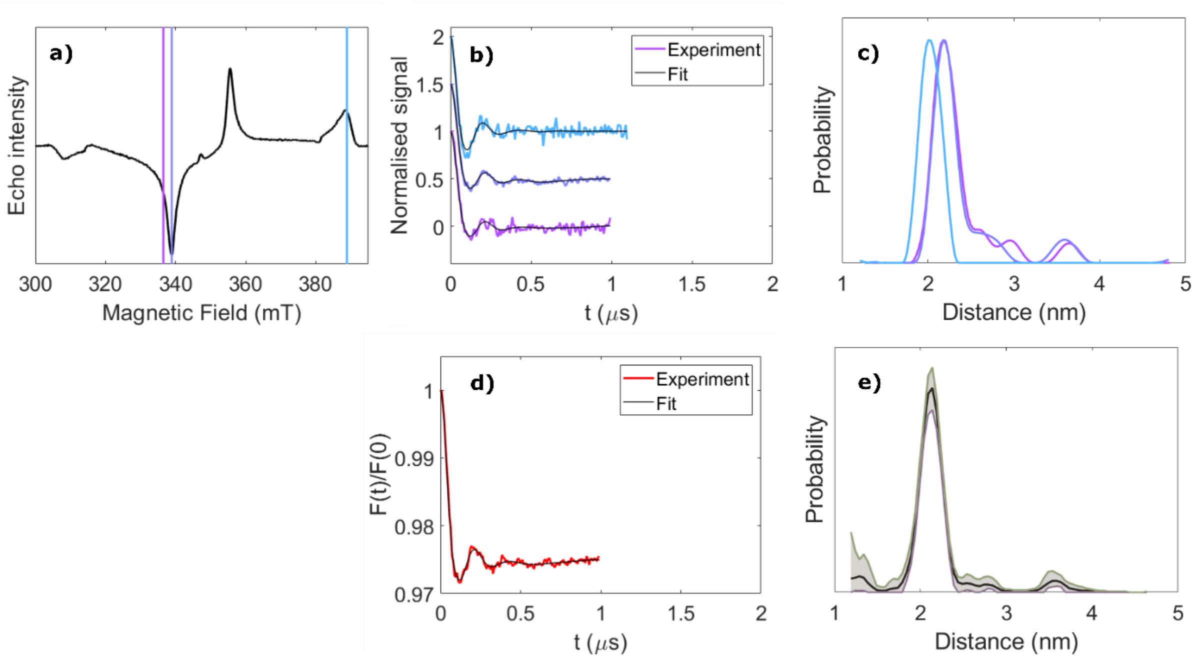


**Figure S22.** Orientation-independent analysis of LITTER with [**1**]. (a) Echo-detected field-swept ESR spectrum of [**1**] measured after 512 nm laser flash. The field positions used to record the LITTER traces are indicated with vertical lines. (b) Background-corrected and modulation depth-normalised LITTER traces acquired at the different field positions (colour) and corresponding orientation-independent fits by Tikhonov regularization using DeerAnalysis[2] (black), $\alpha = 10$. (c) Spin–spin distance distributions obtained from the analysis shown in (b). (d) Averaged LITTER trace (red) and corresponding orientation-independent fit by Tikhonov regularization (black). (e) Spin–spin distance distributions obtained from the analysis shown in (d). 95% confidence bounds have been estimated using the Comparative Deer Analyzer in DeerAnalysis2022 and are shown in gray .[3,4]

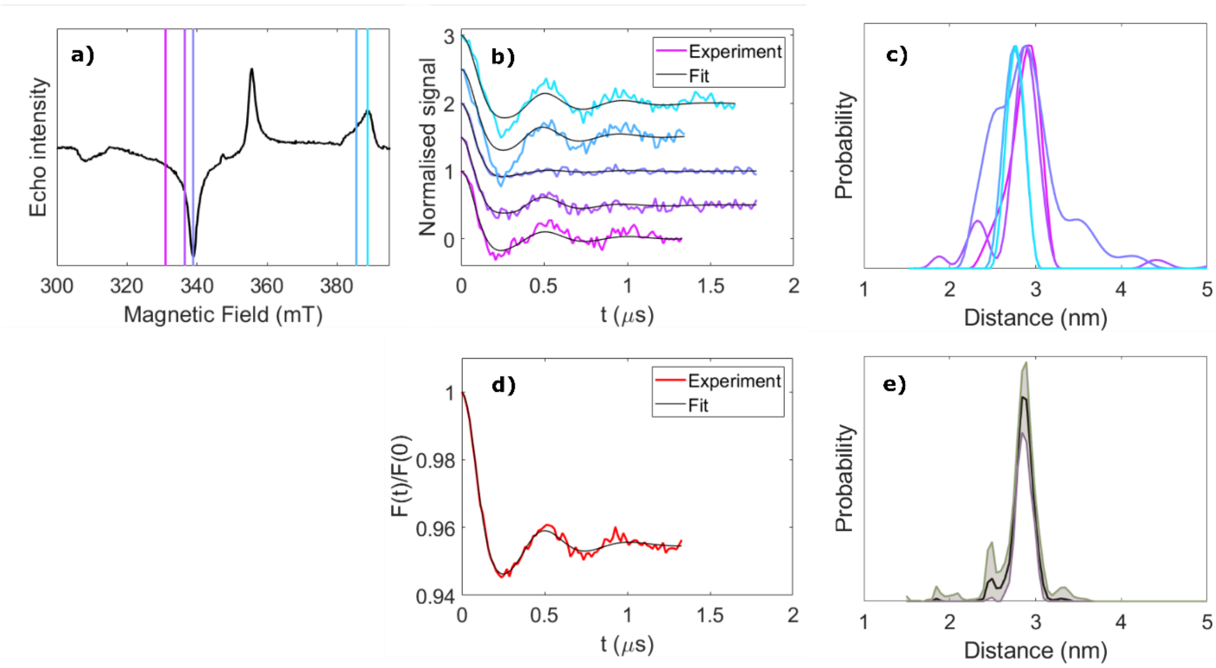


**Figure S23.** Orientation-independent analysis of LITTER with [**2**]. (a) Echo-detected field-swept ESR spectrum of [**2**] measured after 512 nm laser flash. The field positions used to record the LITTER traces are indicated with vertical lines. (b) Background-corrected and modulation depth-normalised LITTER traces acquired at the different field positions (colour) and corresponding orientation-independent fits by Tikhonov regularization using DeerAnalysis[2] (black), $\alpha = 10$. (c) Spin–spin distance distributions obtained from the analysis shown in (b). (d) Averaged LITTER trace (red) and corresponding orientation-independent fit by Tikhonov regularization (black). (e) Spin–spin distance distribution obtained from the analysis shown in (d). 95% confidence bounds have been estimated using the Comparative Deer Analyzer in DeerAnalysis2022 and are shown in gray.[3,4]

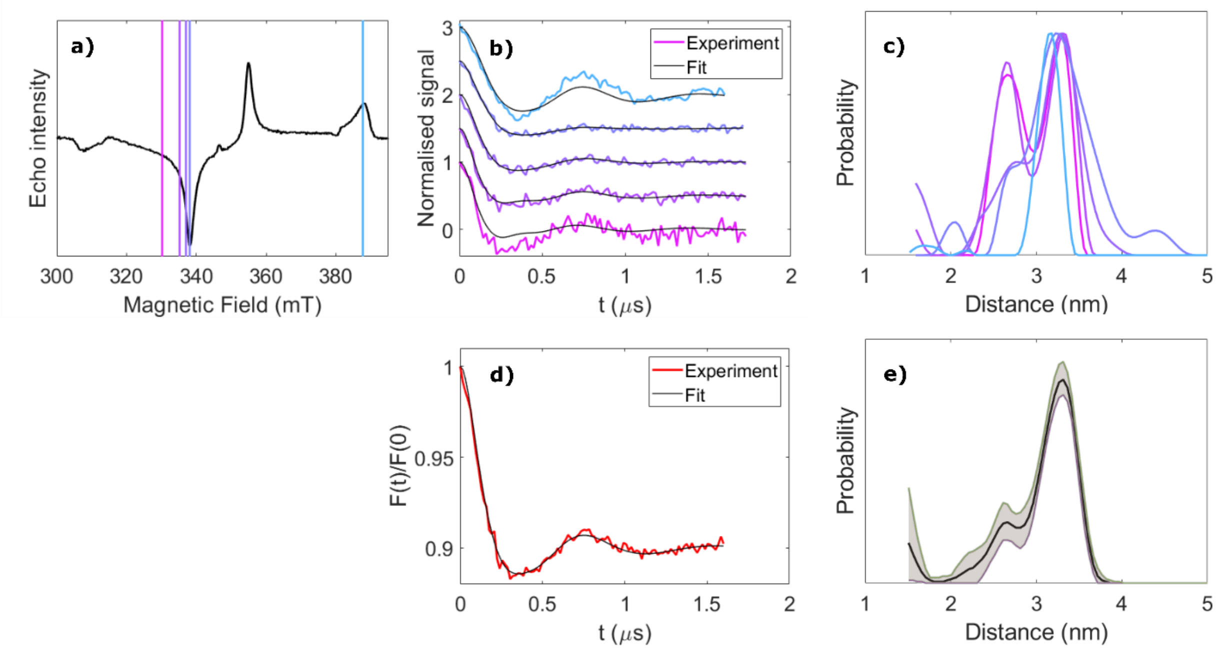


**Figure S24.** Orientation-independent analysis of LITTER with [**4**]. (a) Echo-detected field-swept ESR spectrum of [**4**] measured after 512 nm laser flash. The field positions used to record the LITTER traces are indicated with vertical lines. (b) Background-corrected and modulation depth-normalised LITTER traces acquired at the different field positions (colour) and corresponding orientation-independent fits by Tikhonov regularization using DeerAnalysis[2] (black), $\alpha = 10$. (c) Spin–spin distance distributions obtained from the analysis shown in (b). (d) Averaged LITTER trace (red) and corresponding orientation-independent fit by Tikhonov regularization (black). (e) Spin–spin distance distribution obtained from the analysis shown in (d). 95% confidence bounds have been estimated using the Comparative Deer Analyzer in DeerAnalysis2022 and are shown in gray.[3,4]

To verify the orientation independent methodology used above we have performed simulations on the selected theoretical orientations of the TPP triplet centers simulating traces across the TPP spectrum and comparing the analysis of the sum of all traces to the analysis of only the traces recoded on the Y and Z transitions.

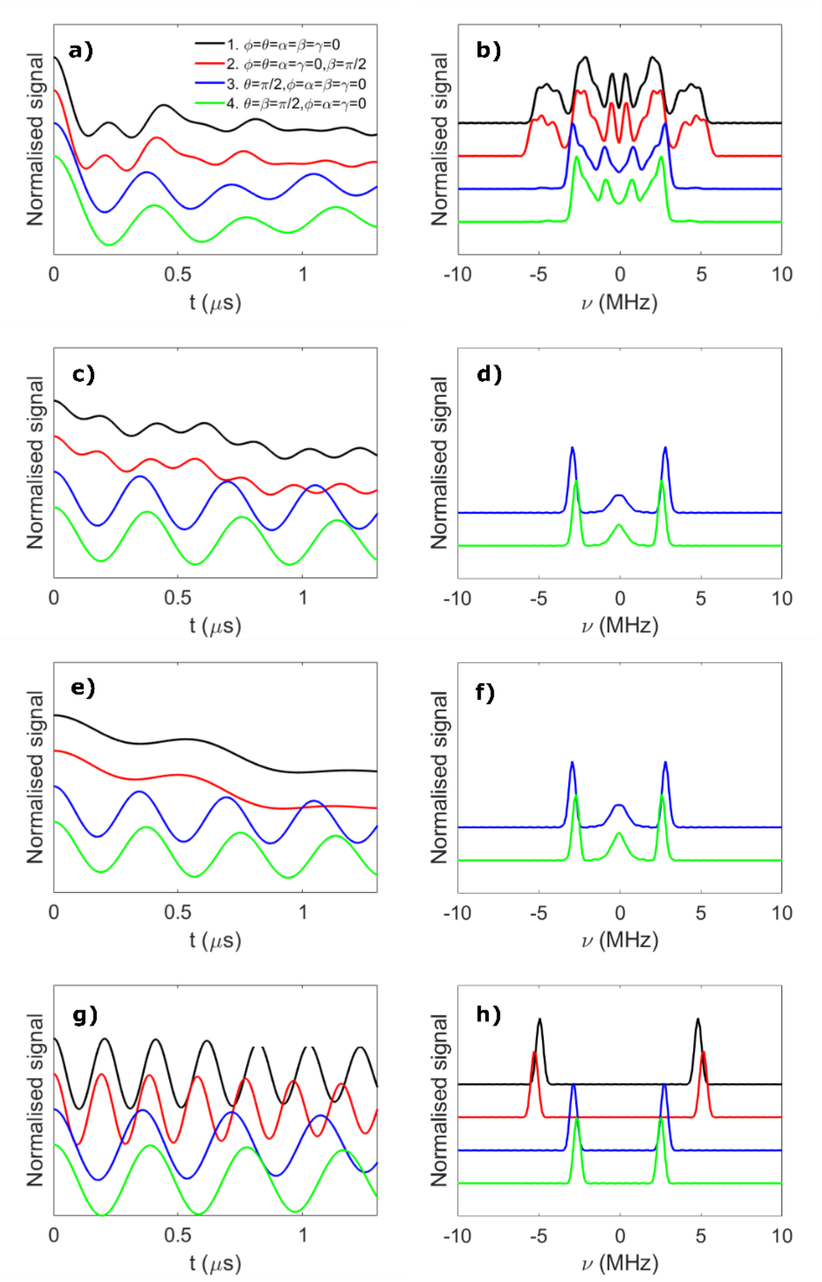

**Figure S25.** Orientational averaging of simulated LITTER traces for selected theoretical relative orientations of the two TPP centres in system [**3**]. (a) Completely averaged traces over the full porphyrin triplet spectrum and (b) corresponding dipolar spectra. (c) Average of only two traces simulated at the field values corresponding to the $Y^-$ and $Z^-$ turning points of the porphyrin triplet spectrum and (d) corresponding dipolar spectra. The traces and dipolar spectra for the individual field values corresponding to the $Y^-$ and $Z^-$ turning points are shown in (e,f) and (g,h), respectively. The selected orientations are given by the centre-to-centre vector between the two TPP moieties $(\theta, \phi)$ and the Euler angles between the ZFS-frames of the two triplet states $(\alpha, \beta, \gamma)$. The following four sets of values were used for the simulations: $\phi = \theta = \alpha = \beta = \gamma = 0$ (black); $\phi = \theta = \alpha = \gamma = 0,\ \beta = \frac{\pi}{2}$ (red); $\theta = \frac{\pi}{2}, \phi = \alpha = \beta = \gamma = 0$ (blue); $\theta = \beta = \frac{\pi}{2}, \phi = \alpha = \gamma = 0$ (green). The porphyrin centre-to-centre distance value was taken from the DFT-optimised structure of [**3**] shown in Fig. 1 c (2.6 nm). Simulations were performed using the orientation-dependent algorithm described in the Experimental Section of the main text. The individual LITTER traces (not shown) were simulated every 5 mT across the porphyrin triplet ESR spectrum. The traces were averaged weighted by the spectral intensities obtained from trESR (Fig. S2). Modulation depths are normalised.

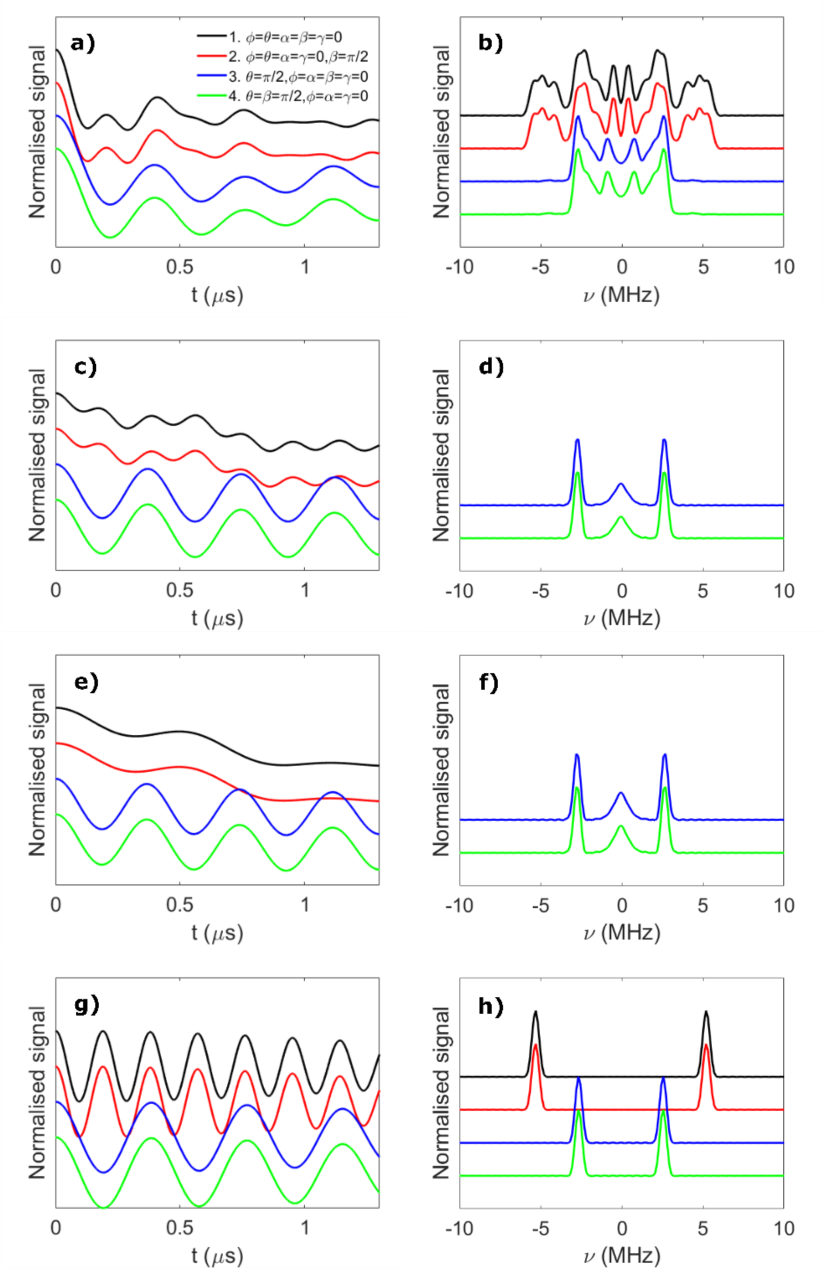


**Figure S26.** Same as Figure S22 but simulated using a single point of spin density at the center of each porphyrin, instead of the triplet spin density delocalized over several atoms as calculated by DFT.

We have further verified this analysis by taking the fitted conformational distribution for system [**4**] which showed the most deviation from the predicted DFT structure and simulated traces across the complete TPP triplet spectrum and compared the analysis of the sum of all of these traces with those only recoded on the Y and Z transitions.

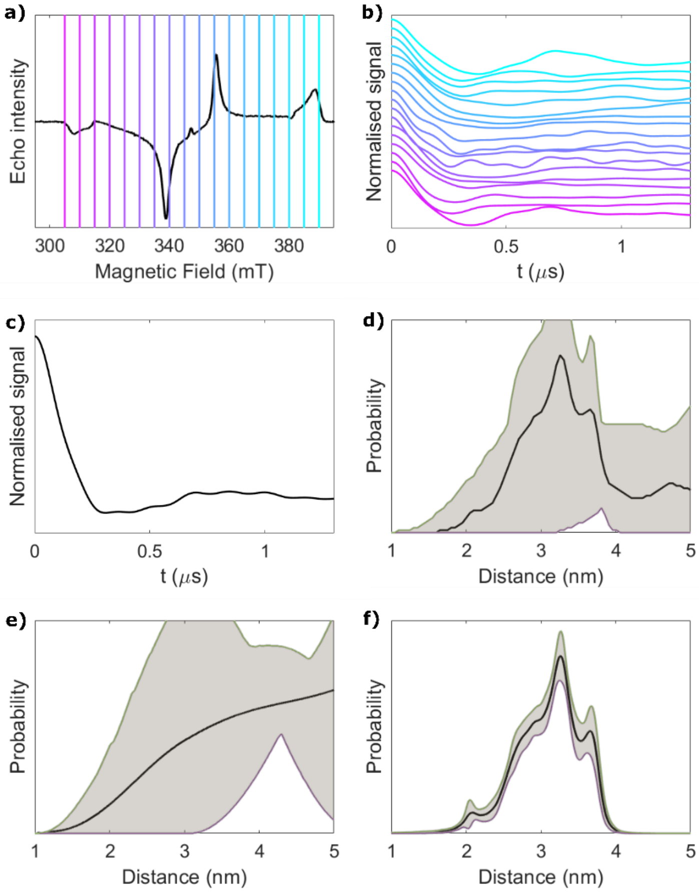


**Figure S27.** (a) Echo-detected field-swept spectrum of [**4**] after photoexcitation and fields used for the simulation. (b) Simulated LITTER traces with the conformational distribution obtained from the fits to the LITTER dataset shown in Fig. 2 d ii. Modulation depths are normalised. (c) Orientationally averaged LITTER trace obtained by summing traces in b weighted by the spectral intensities in a. (d) Spin–spin distance distribution obtained from the analysis of c using the Comparative Deer Analyzer in DeerAnalysis2022 showing the 95% confidence interval in gray.[3,4] (e,f) Spin-spin distance distributions obtained from DeerLab and DeerNet, respectively, using for the confidence bound estimation in Comparative Deer Analyzer. Each distribution shows the 95% confidence interval from each method in gray.

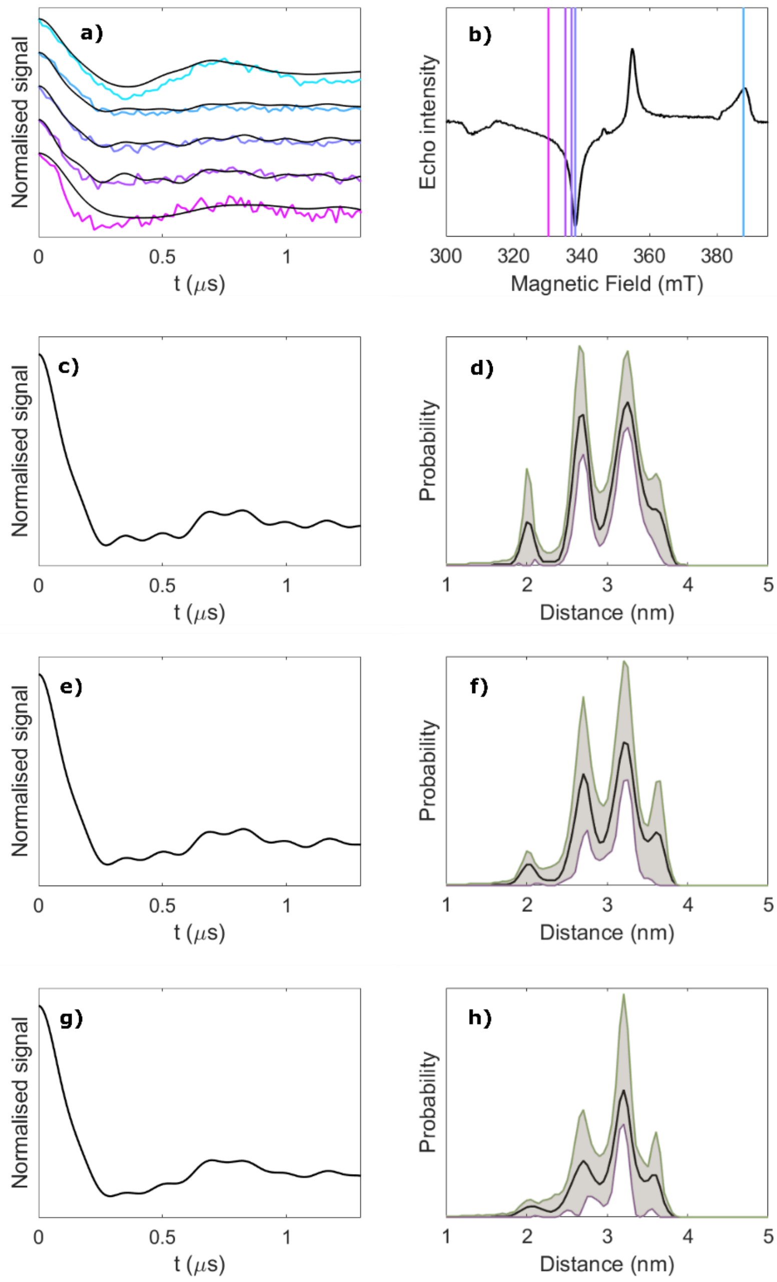


**Figure S28.** (a) Experimental LITTER traces (colours) of [**4**] and simulated LITTER traces (black) of with the conformational distribution obtained from the fits to the LITTER dataset shown in Fig. 2 d ii. Modulation depths are normalised. (b) Echo-detected field-swept spectrum after photoexcitation. The fields used for the experiments and simulations are indicated with vertical lines. (c,d) Average of traces 2, 3, 4 and 5 (from left to right) weighted by the spectral intensities

in b, and corresponding distance distribution. (e,f) Average of traces 3, 4 and 5 weighted by the spectral intensities in b, and corresponding distance distribution. (g,h) Average of traces 4 (Y) and 5 (Z) weighted by the spectral intensities in b, and corresponding distance distribution. Spin-spin distance distributions and 95% confidence bounds (gray) have been obtained using the Comparative Deer Analyzer in DeerAnalysis2022.[3,4]

The same study has been carried out for system [**3**].

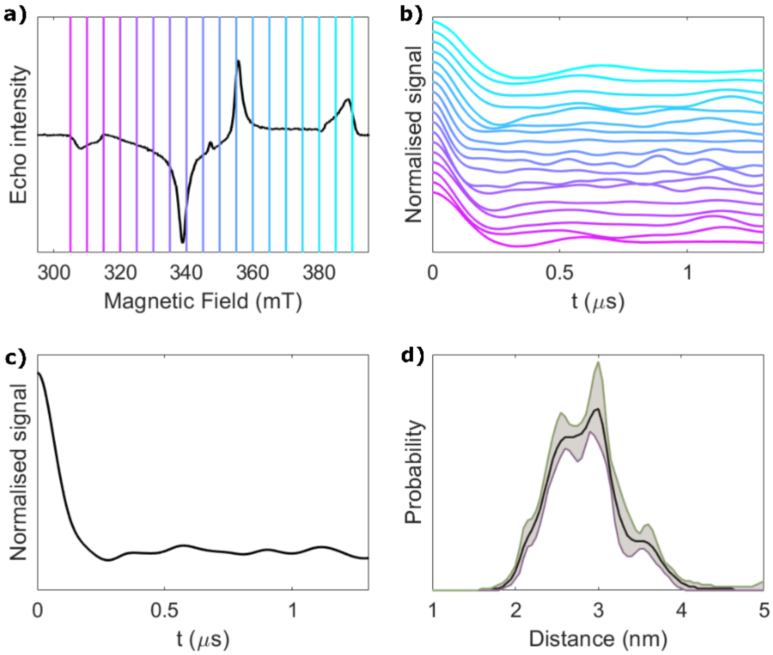


**Figure S29.** (a) Echo-detected field-swept spectrum of [**3**] after photoexcitation and fields used for the simulation. (b) Simulated LITTER traces with the conformational distribution obtained from the fits to the LITTER dataset shown in Fig. 2 c ii. Modulation depths are normalised. (c) Orientationally averaged LITTER trace obtained by summing traces in b weighted by the spectral intensities in a. (d) Spin–spin distance distribution obtained from the analysis of c using the Comparative Deer Analyzer in DeerAnalysis2022 showing the 95% confidence interval in gray.[3,4]

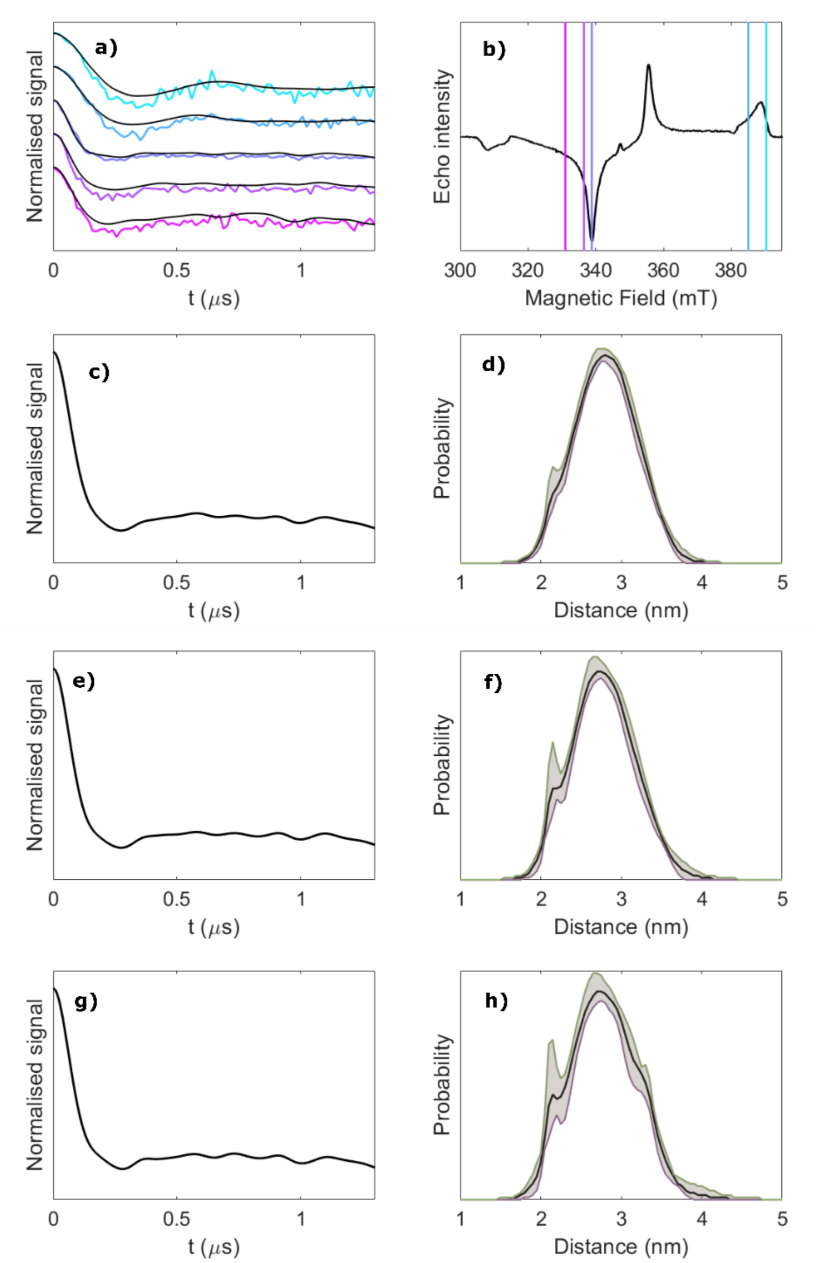


**Figure S30.** (a) Experimental LITTER traces (colours) of [**3**] and simulated LITTER traces (black) of with the conformational distribution obtained from the fits to the LITTER dataset shown in Fig. 2 c ii. Modulation depths are normalised. (b) Echo-detected field-swept spectrum after photoexcitation. The fields used for the experiments and simulations are indicated with vertical lines. (c,d) Average of all traces weighted by the spectral intensities in b, and corresponding

distance distribution. (e,f) Average of traces 2, 3 and 5 (from left to right) weighted by the spectral intensities in b, and corresponding distance distribution. (g,h) Average of traces 3 (Y) and 5 (Z) weighted by the spectral intensities in b, and corresponding distance distribution. Spin-spin distance distributions and 95% confidence bounds (gray) have been obtained using the Comparative Deer Analyzer in DeerAnalysis2022.[3,4]

## S6. Modulation depth to noise ratio (MNR)

Modulation depth to noise ratio is used as a measure of signal quality in pulsed dipolar spectroscopy (PDS) datasets. It is calculated for a trace as the ratio of the modulation depth (varying from 0 to 1) to the noise level of a trace measured as the standard deviation of the experimental trace to a fitted line after the oscillations have been fully dampened. This value is reported for all experimentally recorded traces below.

**Table S4.** Calculation of the modulation depth to noise ratio (MNR) for the dipolar traces reported in this study.

| Experiment/ System | Magnetic field (mT) | Length of trace (μs) | Scans | Square root of scans | Modulation depth | Noise level (stdev) | MNR of recorded trace | MNR normalized by square root of number of scans |
|---|---|---|---|---|---|---|---|---|
| LITTER/[1] | 336.5 | 1.00 | 33000 | 181.66 | 0.029 | $1.1x10^{-3}$ | 27 | 0.148 |
| | 338.9 | 1.00 | 10890 | 104.35 | 0.028 | $4.5x10^{-4}$ | 63 | 0.603 |
| | 388.9 | 1.10 | 31100 | 176.35 | 0.027 | $9.7x10^{-4}$ | 28 | 0.159 |
| LITTER/[2] | 331.0 | 1.30 | 34940 | 186.92 | 0.057 | $2.0x10^{-3}$ | 29 | 0.155 |
| | 336.5 | 1.74 | 7700 | 87.75 | 0.055 | $2.8x10^{-3}$ | 20 | 0.228 |
| | 338.9 | 1.74 | 4750 | 68.92 | 0.047 | $1.4x10^{-3}$ | 33 | 0.479 |
| | 385.5 | 1.30 | 30450 | 174.50 | 0.053 | $1.5x10^{-3}$ | 35 | 0.201 |
| | 388.7 | 1.62 | 8850 | 94.07 | 0.049 | $3.3x10^{-3}$ | 15 | 0.159 |
| LITTER/[3] | 331.0 | 1.31 | 14390 | 119.96 | 0.042 | $2.4x10^{-3}$ | 17 | 0.142 |
| | 336.5 | 1.73 | 8550 | 92.47 | 0.051 | $1.9x10^{-3}$ | 27 | 0.292 |
| | 338.8 | 1.92 | 4000 | 63.25 | 0.053 | $2.4x10^{-3}$ | 22 | 0.347 |
| | 385.0 | 1.34 | 19870 | 140.96 | 0.052 | $3.2x10^{-3}$ | 16 | 0.114 |
| | 390.3 | 1.92 | 18550 | 136.20 | 0.089 | $1.1x10^{-2}$ | 8 | 0.059 |
| LITTER/[4] | 330.2 | 1.73 | 27650 | 166.28 | 0.124 | $1.2x10^{-2}$ | 11 | 0.066 |
| | 335.2 | 1.73 | 13660 | 116.87 | 0.104 | $4.0x10^{-3}$ | 26 | 0.222 |
| | 337.0 | 1.73 | 2590 | 50.89 | 0.102 | $2.6x10^{-3}$ | 39 | 0.766 |
| | 338.1 | 1.73 | 2000 | 44.72 | 0.108 | $3.4x10^{-3}$ | 31 | 0.693 |
| | 387.7 | 1.60 | 13070 | 114.32 | 0.119 | $6.6x10^{-3}$ | 18 | 0.157 |
| DEER/Cu2-[3] | 1144.4 | 0.90 | 11024 | 105.00 | 0.008 | $1.0x10^{-3}$ | 8 | 0.076 |
| | 1156.0 | 0.90 | 7168 | 84.66 | 0.011 | $9.0x10^{-4}$ | 12 | 0.142 |
| | 1165.0 | 0.95 | 6144 | 78.28 | 0.014 | $9.4x10^{-4}$ | 15 | 0.191 |
| | 1176.5 | 0.98 | 7936 | 89.08 | 0.024 | $6.2x10^{-4}$ | 39 | 0.438 |
| | 1183.7 | 1.60 | 3584 | 59.86 | 0.031 | $9.5x10^{-4}$ | 33 | 0.551 |

*Calculated using the background-corrected and normalized traces.

When comparing the MNR of two datasets it is important to note that this value will improve the number of scans or time for which the experiment is recorded. Therefore, if we want to directly compare datasets it is important that we compare the MNR with the number of scans recorded.

## S7. Reporting checklist and data deposition checklist for LITTER

Reporting Checklist for LITTER

- Data confirming the identity, purity, labelling efficiency, and structural/functional integrity of crucial and selected mutants.
- Sample conditions, e.g. concentration, tube size, sample volume, amount and type of cryoprotectant, freezing procedure, deuteration.
- Whether diamagnetic dilution was used along with the way the mixture has been prepared.
- Laser wavelength, power and delivery method(s).
- Spectrometer and resonator.
- Measurement temperature.
- Pulse lengths of detection pulses.
- Positions of pulses with respect to the EPR spectra.
- Parameters describing the shape of the pulses (if they are not rectangular).
- The delays used ($\tau1$ and $\tau2$), shot repetition time, the time increment $\Delta t$ in the primary data, accumulation time, total number of averages (i.e., echoes per point).
- Procedure for reducing orientation selection, if any.
- Measures to reduce multi-spin effects, if any.
- Modulation depth, while noting that this can be variable between experiments due to fluctuations in laser power and samples conditions.
- Signal-to-noise ratio with respect to modulation depth.
- Time offset t0 of the zero time of dipolar evolution in the primary data if a refocused echo sequence is used, otherwise a description of how the zero time was extracted e.g. from symmetrisation of the data recorded using a Hahn echo sequence should be included.
- Equation for a custom kernel, if applicable.
- Information on global fitting of several data sets, if applicable.
- Software used for distance distribution analysis, including version number.
- Regularization parameter and criterion for its selection, if regularization was used.
- Prior information used to constrain the distance distribution.

Data Deposition Checklist

- Primary data as measured, preferably with quadrature detection.
- Distance distribution, including upper and lower limit of the 95% confidence interval. The confidence interval is only expected for averaged traces analysed using software where this feature is included.
- Fit of the primary data, if one-step analysis (preferred) was used – this should only be performed on averaged datasets.
- Background-corrected data V(intra) and their fit, if two-step analysis was used – this is the expected analysis procedure for LITTER datasets particularly as the background can vary from exponential due to relaxation changes as the number of spin active species in the system changes when the laser pump pulse occurs.
- Log file of the data analysis software, if any; otherwise, a list of the settings of the software or processing script.
- If data is not deposited, it should be archived locally and provided upon reasonable request. In addition, all these data should be documented in main text or supplementary figures.

REFERENCES


(1) Stoll, S.; Schweiger, A. EasySpin, a Comprehensive Software Package for Spectral Simulation and Analysis in EPR. *J. Magn. Reson.* **2006**, *178* (1), 42–55. https://doi.org/10.1016/j.jmr.2005.08.013.

(2) Jeschke, G.; Chechik, V.; Ionita, P.; Godt, A.; Zimmermann, H.; Banham, J.; Timmel, C. R.; Hilger, D.; Jung, H. DeerAnalysis2006 - A Comprehensive Software Package for Analyzing Pulsed ELDOR Data. *Appl. Magn. Reson.* **2006**, *30* (3–4), 473–498. https://doi.org/10.1007/BF03166213.

(3) Worswick, S. G.; Spencer, J. A.; Jeschke, G.; Kuprov, I. Deep Neural Network Processing of DEER Data. *Sci. Adv.* **2018**, *4* (8), 1–18. https://doi.org/10.1126/sciadv.aat5218.

(4) Ibáñez, L. F.; Jeschke, G.; Stoll, S. DeerLab: A Comprehensive Software Package for Analyzing Dipolar Electron Paramagnetic Resonance Spectroscopy Data. *Magn. Reson.* **2020**, *1* (2), 209–224. https://doi.org/10.5194/mr-1-209-2020.
